# Grow p‐type MoS_2_ on FeNC for CO_2_ Sensing in Complex Environments with Intelligent Recognition

**DOI:** 10.1002/advs.202512595

**Published:** 2025-10-24

**Authors:** Yuefeng Gu, Yuhao Wang, Jing Ai, Gongjie Liu, Sadaf Saeedi Garakani, Lisi Wei, Zeen Wu, Jiayin Yuan, Qiuhong Li

**Affiliations:** ^1^ School of Electronic Science and Engineering Xiamen University Xiamen 361005 China; ^2^ Department of Chemistry Stockholm University Stockholm 10691 Sweden; ^3^ Division of Chemical Engineering Chalmers University of Technology Gothenburg 41296 Sweden

**Keywords:** CO_2_ sensing, cross‐sensitivity, gas prediction, machine learning, P‐type MoS_2_

## Abstract

A wealth of theoretical studies demonstrates p‐type MoS_2_ (p‐MoS_2_) as a promising candidate for carbon dioxide (CO_2_) detection at room temperature. Its applications are retarded by issues associated with its practical chemical synthesis and sensing selectivity. Herein, a chemically tunable strategy is established for in situ growth of p‐MoS_2_ with controlled thickness and n‐/p‐type transition on N‐ and Fe‐enriched carbon (FeNC) nanosheets. The introduced sulfur vacancies (S_vacs_) enhance the sensitivity to CO_2_, and the modulated electron distribution suppresses surface oxygen ionization to improve sensing selectivity. The optimized p‐type composites can detect CO_2_ fluctuation levels as low as 50 ppm at room temperature. Density functional theory (DFT) and grand canonical Monte Carlo (GCMC) simulations clarify the underlying mechanisms. A visualized machine learning (ML) model is developed using a hybrid ML strategy that generates regression surfaces from linear/nonlinear data. Through this model, a single sensor accurately discriminates CO_2_ from interfering and predicts its concentration and humidity with accuracies exceeding 95%. An intelligent sensing system capable of environmental monitoring and tracking exhaled CO_2_ is demonstrated. The measured fluctuations strongly correlate with physiological indicators, underscoring their potential for non‐invasive health monitoring and medical diagnostics.

## Graphical abstract

In situ growth of p‐type MoS_2_ (p‐MoS_2_) on N‐ and Fe‐enriched carbon (FeNC) nanosheets allows for detection of carbon dioxide (CO_2_) fluctuation levels as low as 50 ppm at 25 °C. The induced S vacancies (S_vacs_) and the modulated electron distribution jointly improved sensing selectivity to CO_2_.

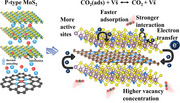

## 1 Introduction

Over the past decades, continuously increasing fossil fuel emissions have led to a rapid rise in atmospheric carbon dioxide (CO_2_) concentrations.^[^ ^1^, ^2^ ^]^ Observations indicate that CO_2_ levels have increased from ≈280 ppm to over 421 ppm today since the industrial revolution.^[^ ^3^ ^]^ The ever‐increasing greenhouse gas emissions, particularly CO_2_, have accelerated global warming and intensified the risk of extreme, potentially irreversible climate change.^[^ ^4^, ^5^ ^]^ While international consensus has underscored the urgency of curbing greenhouse gas emissions, the spatial heterogeneity of CO_2_ distribution posed a significant challenge to the effective implementation of mitigation strategies. For instance, CO_2_ concentrations in industrial zones were markedly higher than in other areas. The heavy manufacturing facilities emitted significantly more CO_2_ than the light industry. Therefore, long‐term monitoring of CO_2_ concentrations was essential for tracking emission sources. Such monitoring served not only to identify major emission contributors and guide industrial zoning and policy decisions but also to facilitate the strategic placement of carbon capture infrastructure. In addition, indoor CO_2_ monitoring has gained increasing importance for public health.^[^ ^6^, ^7^ ^]^ Concentrations exceeding 1000 ppm in enclosed environments were known to impair cognitive performance and induce physiological symptoms such as fatigue, respiratory irritation, and headaches.^[^ ^8^ ^]^ CO_2_ detection has also shown significant potential in biomedical applications, as exhaled CO_2_ levels serve as a non‐invasive proxy for metabolic and respiratory function, with implications for health assessment and disease diagnosis.^[^ ^9^ ^]^ Correlations have been reported between exhaled CO_2_ and blood glucose levels, and disease‐specific variations in breath CO_2_ concentrations have been observed. These findings underscored the critical need for sensitive, selective, and real‐time CO_2_ sensing technologies across both environmental and healthcare domains.^[^ ^10^ ^]^

Current CO_2_ sensing technologies predominantly rely on metal oxide semiconductors (MOS). For instance, Singh et al. synthesized a ZnO nanoflake‐based CO_2_ gas sensor, which presented a fast response (<20 s) and high sensitivity (0.1125 ppm^−1^ for 600 ppm).^[^ ^11^ ^]^ Joshi et al. reported Ag‐doped CuO/ZnO heterostructures that achieved a 34% response to 1000 ppm CO_2_ at 320 °C.^[^ ^12^ ^]^ However, the weak interaction between MOS and CO_2_ has made it challenging to reduce the operating temperature of these sensors. This limitation often necessitates the use of bulky external heating components to maintain sensor functionality, thereby hindering their integration into compact and portable electronic devices. Strategies like doping and surface modification have been explored, but room temperature (R.T.) sensing remains challenging.^[^ ^13^ ^]^ For example, Zito et al. prepared a yolk‐shell nanostructured CeO_2_ that can detect CO_2_ at a low temperature of 100 °C with the response and recovery time of minutes. Polymer‐based sensors could work at R.T. while suffering from sluggish desorption kinetics and inherently poor electronic conductivity.^[^ ^14^ ^]^

2D materials have emerged as compelling alternatives to conventional MOS for gas sensing applications, owing to their high specific surface area, atomic‐scale thickness, rapid charge transport, and tunable electrical and chemical properties.^[^ ^15^, ^16^ ^]^ Notably, their intrinsic ability to operate at R.T. rendered them particularly attractive for portable and low‐power gas sensing devices.^[^ ^17^ ^]^ Among the various 2D materials, molybdenum disulfide (MoS_2_) has attracted widespread attention due to its semiconducting characteristics, tunable bandgap, abundance of active sites, structural defects, and exposed edge planes.^[^ ^18^, ^19^ ^]^ Luo et al. prepared a V_2_CT_x_/MoS_2_ composite material that exhibited excellent sensitivity to ppb‐level NH_3_ at R.T.^[^ ^20^ ^]^ Huo et al. reported an In_2_O_3_@MoS_2_ heterojunction with a 322.1‐fold higher response to H_2_S than the In_2_O_3_ sensor at R.T.^[^ ^21^ ^]^ However, most experimental studies on MoS_2_‐based sensors have focused on polar gases such as NO_2_, NO, and H_2_S. Experimental reports on CO_2_ sensing remain limited, with most insights derived from theoretical calculations, and there have been no experimental studies on p‐type MoS_2_ in this field.^[^ ^22^, ^23^, ^24^ ^]^ This largely arose from the unresolved challenge of achieving satisfactory selectivity. Theoretical predictions indicated strong CO_2_ adsorption on MoS_2_, with p‐type MoS_2_ (p‐MoS_2_) showing enhanced development potential.^[^ ^25^, ^26^ ^]^ Shokri et al. demonstrated that MoS_2_ monolayers have good adsorption capability for chemically active small molecules (CO, CO_2_, and NO) through the density functional theory (DFT) calculation.^[^ ^27^ ^]^ Francis et al. proved through calculations that N‐doped MoS_2_ exhibited enhanced and selective CO_2_ adsorption.^[^ ^28^ ^]^ Kim et al. found that MoS_2_ presents changing sensitivity to different gases as the number of layers changes, with optimal sensitivity observed in few‐layer configurations. However, the excessive layer thickness suppresses gas interaction while ultra‐thin films were prone to performance fluctuations.^[^ ^29^ ^]^ At present, most strategies focused on modifying the properties of n‐type MoS_2_ (n‐MoS_2_) through the introduction of p‐type dopants. However, conventional techniques such as ion implantation and physical deposition were both cost‐intensive and required stringent processing conditions. Although chemical doping approaches were more accessible, they often lacked precise control over dopant concentrations. Consequently, the development of an effective and controllable chemical doping strategy to fabricate p‐MoS_2_ remained essential.

Another critical challenge was the complexity of real‐world working environments, including interfering gases and humidity variations. Despite substantial research efforts to address cross‐sensitivity under complex atmospheric conditions, the wide diversity of gas species resulted in highly intricate interactions. Fortunately, artificial intelligence (AI) powered statistical data analysis methods have brought a breakthrough. Machine learning (ML) techniques based on sensor response or electrical signal analysis have been widely adopted for multi‐gas identification.^[^ ^6^ ^]^ While this approach effectively addressed selectivity challenges, the training outcomes heavily depended on the material's signal response variations toward different gas species. Moreover, achieving accurate concentration prediction across diverse environmental conditions typically required multiple sensors and complex calibration procedures, significantly increasing costs. Therefore, an ideal solution would be a single sensor capable of both target gas identification and predictive functionality. In summary, the challenges for MoS_2_‐based R.T. CO_2_ sensors could be categorized into two key aspects: 1) A controllable chemical method for p‐MoS_2_ fabrication; and 2) ML frameworks capable of providing identification and prediction functions with a single sensor.

Herein, a simple strategy was established for constructing R.T. CO_2_ sensors. Initially, vacancy‐rich MoS_2_ was grown on N‐ and Fe‐enriched carbon (FeNC) nanosheets via a high‐temperature pyrolysis method. This approach constituted an “inside‐out” p‐type doping mechanism, wherein N‐ and Fe‐enriched carbon substrates served as dopant reservoirs. Upon thermal annealing, these dopants diffused into the MoS_2_ lattice, inducing a p‐type transition. The precursor ratio can be adjusted to tailor the composite's structural and electronic properties. During this process, Fe primarily induced the formation of sulfur vacancies (S_vacs_), while N substitution for S altered the electron distribution within the MoS_2_, significantly enhancing non‐covalent interactions between the material and CO_2_. Moreover, a significant number of electrons was transferred from MoS_2_ to FeNC, substantially depleting the MoS_2_ electron density. This suppresses oxygen ionization at the surface, thereby minimizing cross‐sensitivity to interfering gases and improving CO_2_ selectivity. Density functional theory (DFT) and Grand canonical Monte Carlo (GCMC) simulations were performed to understand the sensing mechanism. A portable sensor was fabricated using the synthesized composite, and a visualized ML model was developed to resolve complex nonlinearities in the concentration‐response relationship. This hybrid ML framework integrated regression surface modeling with a decision graph in 2D principal component (PC) space, enabling selective identification of CO_2_ in mixed‐gas environments using a single sensor. Finally, the practical viability of the sensor was validated across diverse environmental scenarios.

## 2 Results and Discussion

**Figure 1:**
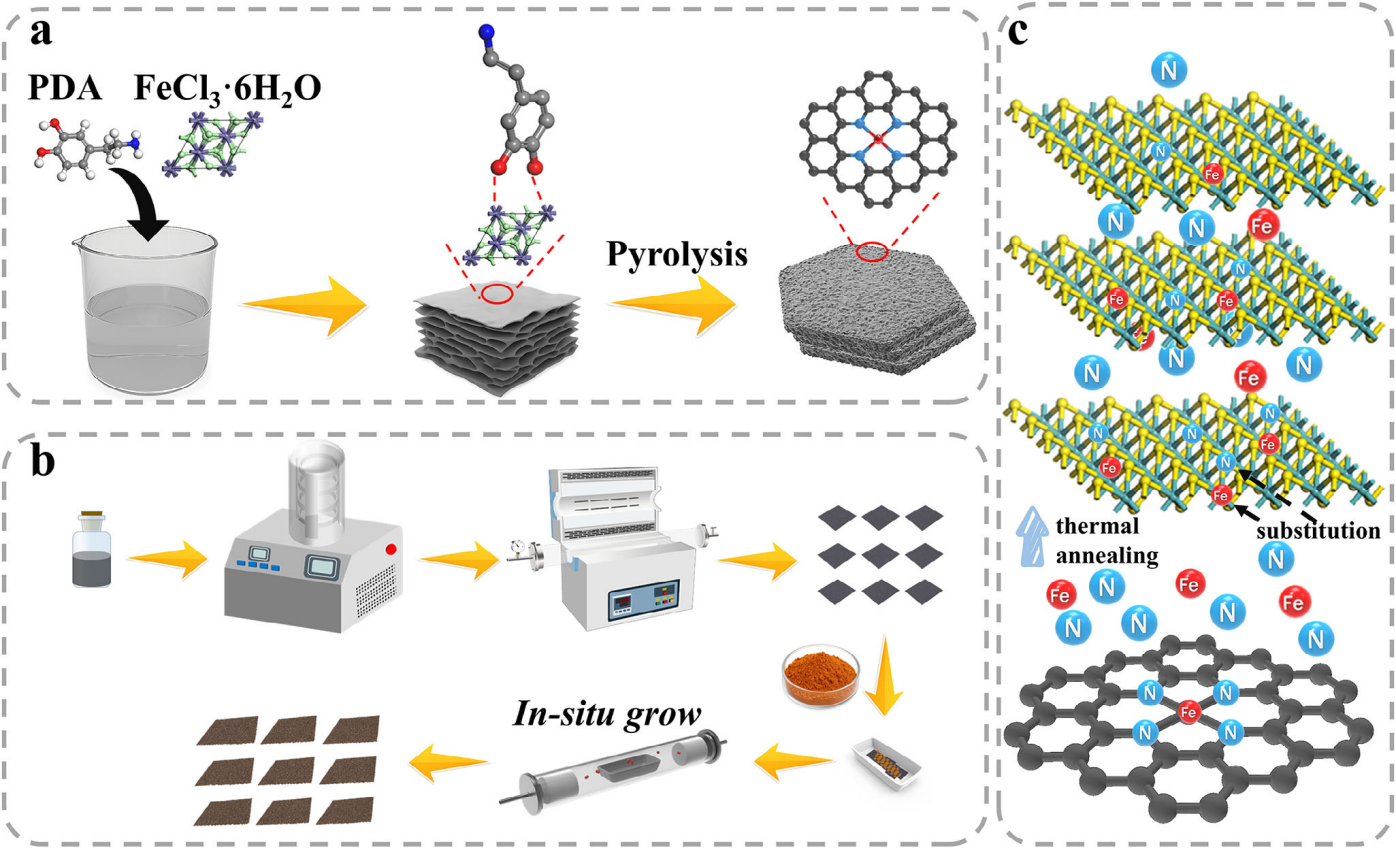
Schematic illustration of the stepwise fabrication of the FeNC/MoS_2_ composite sensor. a) Synthesis of FeNC nanosheets; b) Growth of MoS_2_ on FeNC; c) Migration of Fe/N from FeNC to MoS_2_ under thermal annealing.

First, the potential of FeNC nanosheets as the substrate enriched by p‐type doping elements was evaluated. FeCl_3_·6H_2_O can strongly interact with ‐OH and ‐NH_2_ groups of PDA, inducing a spatially well‐ordered coordination assembly of PDA molecules into layered structure, as reported previously.^[^ ^30^ ^]^ Such layered structures were chosen as scalable, low‐cost, and environmentally benign precursors to FeNC nanosheets. The formed organic‐inorganic hybrid remained morphologically similar after calcination, producing N and Fe‐enriched 2D carbon nanosheets. (**Figure** **1**a). Subsequently, (NH_4_)_2_MoS_4_ was mixed with the as‐synthesized FeNC nanosheets in a controlled mass ratio and subjected to high‐temperature pyrolysis (Figure 1b). The FeNC nanosheets with a porous architecture, high surface area, and abundant active sites facilitated the uniform growth of MoS_2_ layers while suppressing particle agglomeration. Pyrolysis of the FeNC/MoS_2_ composite promoted the diffusion of Fe and N species into the MoS_2_ lattice to modify its local charge distribution and the Mo─S bonds. It facilitated Fe and N to partially substitute Mo and S atoms, respectively, introducing p‐type doping. The mismatch of atomic radius in the host (Mo and S) and guest atoms (Fe and N) further induced lattice distortion, generating abundant vacancies (Figure 1c).

**Figure 2:**
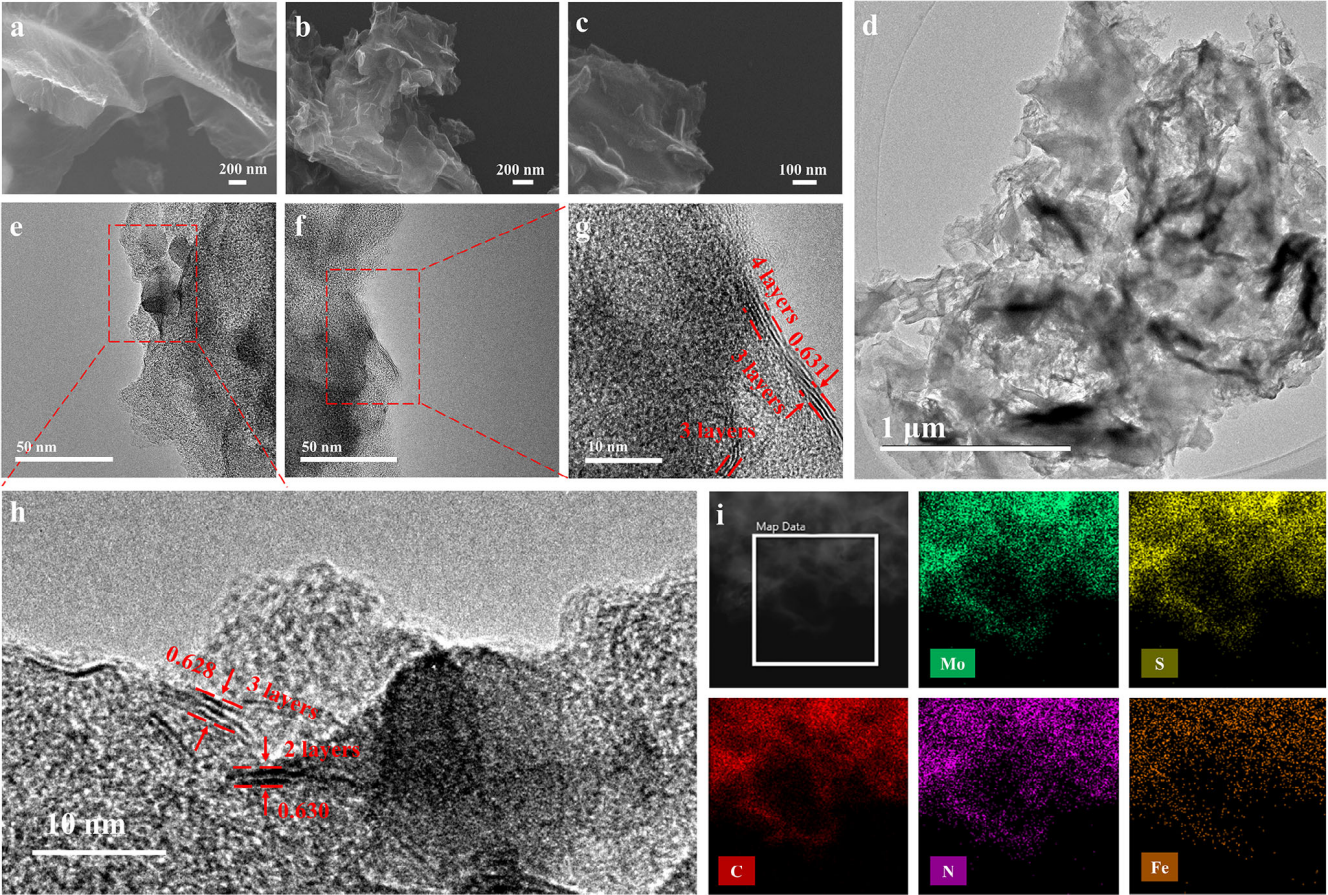
SEM images of a) FeNC and b,c) FeNC/MoS_2_‐1:2 at different magnifications. d‐h) TEM images of FeNC/MoS_2_‐1:2. i) EDX elemental mapping of FeNC/MoS_2_‐1:2, showing the uniform distribution of Mo, S, C, N and Fe.

Scanning electron microscopy (SEM) revealed that the obtained FeNC was in a crumpled, ultrathin sheet‐like morphology with thickness below 15 nm (**Figure** **2**a; Figure S3, Supporting Information). Figure 2b,c, and Figure S4 (Supporting Information) showed that the as‐synthesized FeNC/MoS_2_ retained the sheet‐like structure of pristine FeNC at a FeNC/MoS_2_ precursor mass ratio in the range of 1:0.5 to 1:4. It is seen that when the Mo content increased, the crumpled features became gradually less obvious and eventually disappeared, indicating that thicker MoS_2_ layers prevents the curling of nanosheets due to high mechanical resistance. At an even higher FeNC/MoS_2_ precursor mass ratios of 1:6 and 1:8, the nanosheet morphology was no longer visible (Figure S5a,b, Supporting Information), exhibiting large bulk‐like structures resembling that of MoS_2_ directly obtained through pyrolysis of (NH_4_)_2_MoS_4_ (Figure S5c, Supporting Information). In the transmission electron microscopy (TEM) analysis, at a FeNC/MoS_2_ precursor mass ratio of 1:1, the deposited MoS_2_ phase on FeNC nanosheets primarily formed up to 2 layers with an average spacing d of 0.59 ± 0.02 nm (Figure S6, Supporting Information), while 3–4 layers (d ≈ 0.63 ± 0.02 nm) at a precursor mass ratio of 1:2 (Figure 2d‐h), and beyond 10 layers at a precursor mass ratio of 1:6 (d ≈ 0.65 ± 0.02 nm) (Figure S7, Supporting Information). The change in interlayer spacing was related to doping, and the detailed discussion was presented in the Supporting Information. Elemental mapping (Figure 2i) verified uniform Mo and S distribution across the layered FeNC surface. These observations verify that the FeNC/MoS_2_ precursor mass ratio can readily control the number of MoS_2_ layers grown on FeNC nanosheets.

**Figure 3:**
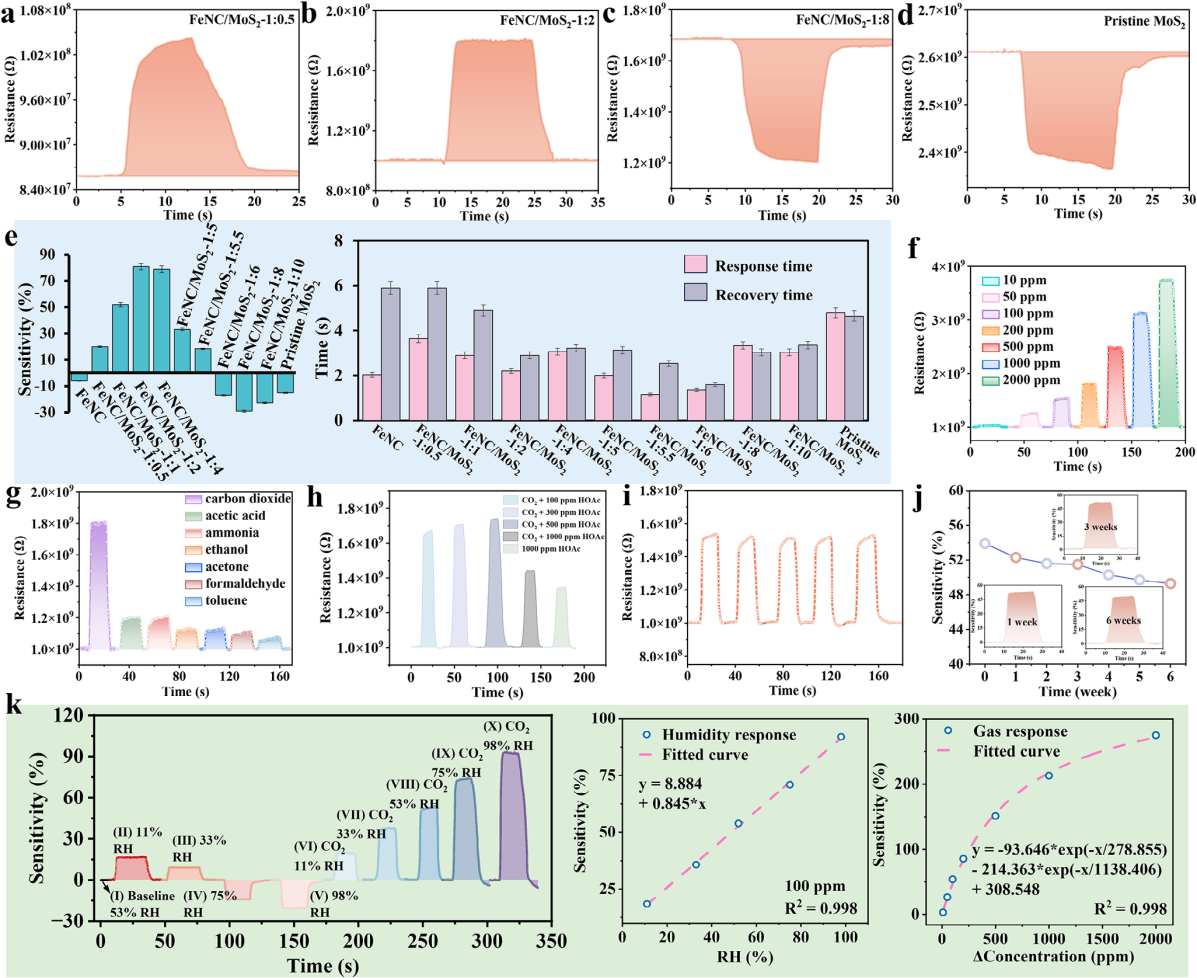
Response curves to 200 ppm CO_2_ of a) FeNC/MoS_2_‐1:0.5, b) FeNC/MoS_2_‐1:2, c) FeNC/MoS_2_‐1:8, d) Pristine MoS_2_, and e) sensitivity and response/recovery times of samples. Response curves of FeNC/MoS_2_‐1:2 f) at different CO_2_ concentrations, g) at different interfering gases at 200 ppm. Response curves to 100 ppm CO_2_ of h) coexisting with different concentrations of acetic acid (HOAc), i) repeatability test, and j) stability test after different weeks. k) Response curves of FeNC/MoS_2_‐1:2 to 100 ppm CO_2_ with different RH, and the relationship between sensitivity and different RH/CO_2_ concentrations.

The response curves of samples at R.T. to CO_2_ were displayed in **Figures** **3** and S8 (Supporting Information). As the MoS_2_ content increased, the response characteristics exhibited notable changes (Figure 3a‐d). Overall, sensitivity increased first and then decreased, peaking at FeNC/MoS_2_‐1:2 with the highest sensitivity of 81%. In comparison, pristine FeNC delivered a low response of ‐6%. FeNC/MoS_2_‐1:0.5 has the lowest MoS_2_ content in all studied samples and also gave a low sensitivity of 20% due to insufficient MoS_2_ coverage on the FeNC surface. As the MoS_2_ content increased from 1:1 to 1:2, the sensitivity improved from 52% to 81%, followed by a slight drop to 79% at a 1:4 precursor mass ratio, likely due to reduced vacancy concentrations and increased electron density at higher MoS_2_ thicknesses (Figure 3e). This effect weakened the interaction between vacancies and CO_2_, thus lowering down sensitivity. Prolonged response and recovery times were ascribed to chemical interactions between CO_2_ and ionized oxygen species. The distinct response behaviors of FeNC and n‐MoS_2_ originated from their different reduction pathways (details in Supporting Information). The MoS_2_ phase produced at a FeNC/MoS_2_ precursor mass ratio in the range of 1:0.5 to 1:5.5 was found to be p‐type. Interestingly, at an even higher MoS_2_ content above 1:5.5, opposite resistance changing behavior was observed, which was consistent with the pristine n‐type 2H‐MoS_2_. As designed, the limited dopant atoms of N and Fe were difficult to diffuse through the increasingly thick MoS_2_ layers, which prevented the newly formed n‐type MoS_2_ from converting to p‐type, thus the composites exhibited n‐type characteristics. As such, FeNC/MoS_2_‐1:2 was selected for further testing due to its highest sensitivity. As shown in Figure 3f,g, FeNC/MoS_2_‐1:2 displayed strong sensitivity to CO_2_ across a range of concentrations, achieving a stable and repeatable detection limit as low as 50 ppm, along with outstanding selectivity toward CO_2_. A minimum fluctuation of 10 ppm was detectable with a sensitivity of 2.96%, as realized under laboratory conditions. Acetic acid (HOAc) and ethanol were chosen as interfering gases for cross‐sensitivity tests. Both exhibited profiles with an initial rise followed by a drop, originating from intensified competitive adsorption at high concentrations (Figure 3h; Figure S9, Supporting Information). The sample exhibited excellent repeatability under varying temperature (Figure S10, Supporting Information), humidity (Figure S11, Supporting Information), and concentration conditions (Figure S12, Supporting Information), and also demonstrated outstanding long‐term stability, with sensitivity fluctuations within 3% after six weeks of continuous testing (Figure 3i,j). The response–recovery times for each cycle were recorded and presented in Figure S13 (Supporting Information), showing an overall increasing trend (details in Supporting Information). Figure S14 (Supporting Information) recorded the temperature‐sensing characteristics under different RH. In the range of 25–100 °C, the resistance changes were nearly linear. When the temperature exceeded 100 °C, the magnitude of the resistance change decreased, which might have been due to the adsorption–desorption behavior of water vapor on the material surface approaching equilibrium within this temperature range. Figure S15 (Supporting Information) showed that the sensitivity fluctuated very little within the R.T. range (25–30 °C). When the temperature increased further, the sensitivity decreased, which was attributed to the suppression of CO_2_ physical adsorption at higher temperatures. Figure 3k presented the sensor sensitivity to humid air and CO_2_ in different RH. Statistical analysis demonstrated the consistent linear response‐humidity relationships across 11–98% RH (Figure 3k; Figure S16, Supporting Information). The correlation between sensitivity and CO_2_ concentration was nonlinear, consistent with Langmuir‐type physical adsorption. To further assess the effect of humidity, tests were conducted using CO_2_‐free synthetic air. Figure S17 (Supporting Information) showed that the influence of humidity alone on the sensitivity did not exceed 9%. However, under standard conditions, humidity affected the sensitivity by up to 20%. This is likely because, in the presence of humidity, interactions occur between CO_2_ and water, including competitive adsorption and other interactions, and this effect is also influenced by temperature (Figure S18, Supporting Information). Therefore, the actual sensor response reflected the combined effect of both factors rather than a simple linear superposition (details in Supporting Information). As shown in **Table** **1**, our material (FeNC/MoS_2_‐1:2) demonstrated superior performance compared to previously reported CO_2_ sensors.

**Table 1:** Comparison of key parameters of this work with other reported CO_2_ sensors.

Sensing materials	T. [°C]	Analyte concentration [ppm]	Response [%]
Graphene/PANI^[^ ^31^ ^]^	R.T.*	50	10
Ti_3_C2T_x_/PANI^[^ ^32^ ^]^	R.T.	500	15.2
TiS_2_ nanodiscs^[^ ^33^ ^]^	R.T.	500	60
Na/In_2_O_3_^[^ ^34^ ^]^	200	400/1200	320/800
CaO‐ZnO^[^ ^35^ ^]^	150	500	77
Au‐ZnO^[^ ^36^ ^]^	250	200	80
ZnO film^[^ ^37^ ^]^	350	400	65
Graphene/PEI/PEG^[^ ^38^ ^]^	R.T.	500	6
P‐Si/Mo_2_CT_x_^[^ ^39^ ^]^	30	100	10
NiO/Cu_x_O^[^ ^40^ ^]^	250	1000	60
rGO‐CuO^[^ ^13^ ^]^	R.T.	500	39.6
Graphene/CsPbBr_3_^[^ ^41^ ^]^	R.T.	50	0.7
Ag@CuO/BaTiO_3_^[^ ^42^ ^]^	120	100	10
PEI/PEG/ Ti_3_C_2_T_x_^[^ ^43^ ^]^	R.T.	2000/10000	200/414
2L MoS_2_^[^ ^29^ ^]^	60	5000	15
This work	R.T.	100	54

*R.T. is room temperature.

**Figure 4:**
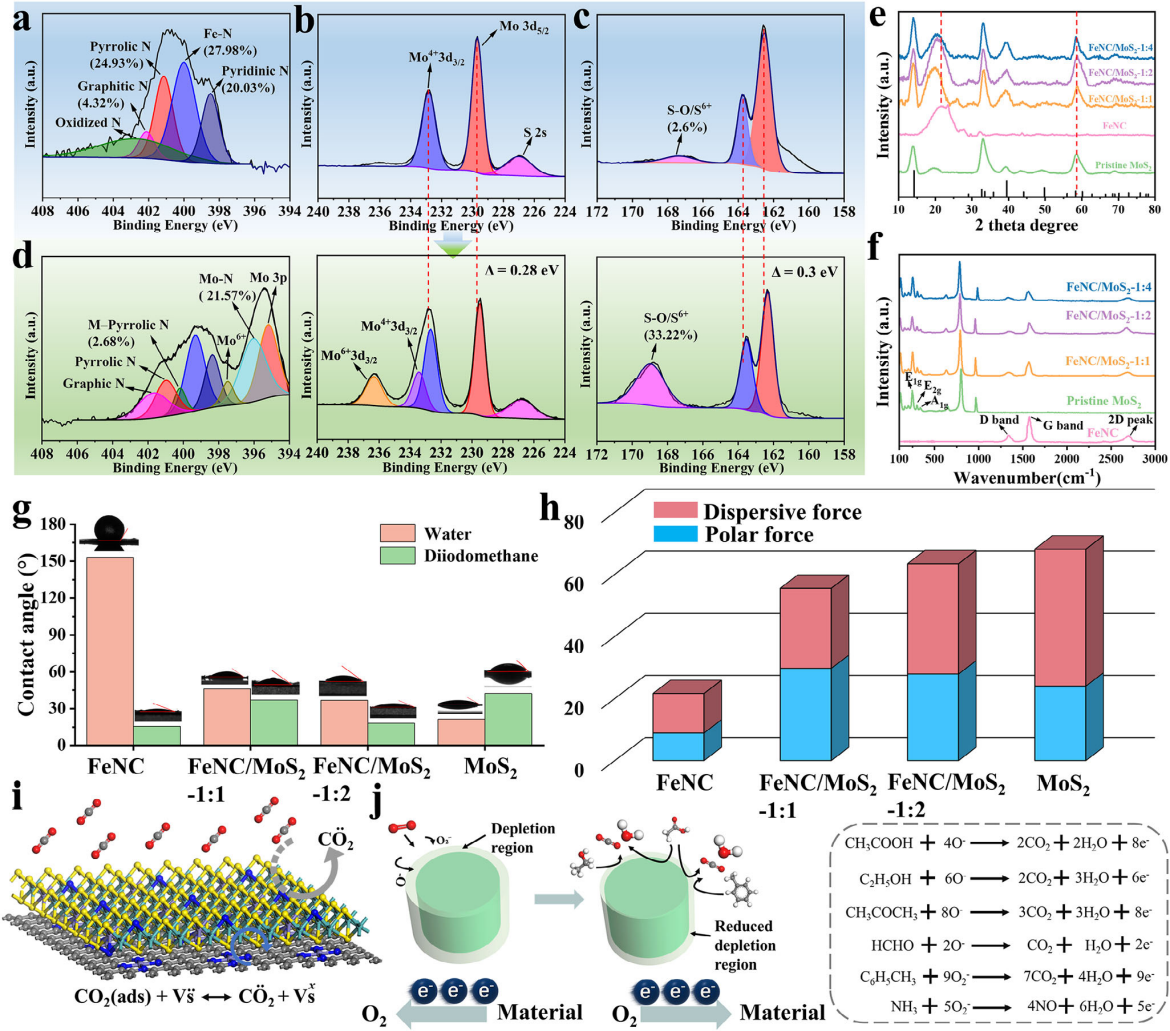
XPS spectra of a) FeNC, b,c) MoS_2_, and d) FeNC/MoS_2_‐1:2. e) XRD pattern (MoS_2_, JCPDS: 27–1492). f) Raman spectra. g) Contact angle. h) Surface energy. i) Schematic reaction diagram between CO_2_ and p‐type FeNC/MoS_2_. j) Sensing mechanism of p‐type FeNC/MoS_2_ in the interfering gases, depletion layer changing of n‐type FeNC/MoS_2_, and detailed reaction steps.

A number of characterization techniques (**Figure** **4**a‐f) were used to reveal the structure of samples. In the X‐ray photoelectron spectroscopy (XPS) N 1s spectrum of FeNC, peaks corresponding to pyridinic N (398.5 eV) of 20.03 wt.%, Fe–N (400.1 eV) of 27.98 wt.%, pyrrolic N (401.2 eV) of 24.93 wt.%, graphitic N (402.1 eV) of 4.32 wt.%, and oxidized N (402.8 eV) were observed (Figure 4a). The Fe 2p spectrum exhibited peaks at 711.4, 716.8, and 725.7 eV, assigned to Fe^2^⁺ 2p_3_/_2_, Fe^3^⁺ 2p_3_/_2_, and Fe^2^⁺ 2p_1_/_2_, respectively (Figure S19a, Supporting Information), indicating chemically bonded Fe rather than metallic Fe⁰. As for MoS_2_, the Mo 3d peaks at 232.8 and 229.7 eV were attributed to Mo 3d_3_/_2_ and Mo 3d_5_/_2_, respectively, with no evidence of split Mo⁴⁺ peaks, indicating the absence of the metallic 1T phase (Figure 4b). The S 2p spectrum showed peaks at 162.4 eV (S 2p_3_/_2_) and 163.7 eV (S 2p_1_/_2_) corresponded to divalent S species, and a minor peak at 167.3 eV (2.6 wt.%) indicating combined states of S species (S–O/S⁶⁺) (Figure 4c). In the sample of FeNC/MoS_2_‐1:2, the N 1s spectrum exhibited a substantial presence of Mo─N bonds (21.57%) and a new Fe‐pyrrolic N coordination peak at 400.2 eV.^[^ ^44^, ^45^ ^]^ The Mo 3d spectrum displayed additional peaks at 233.4 eV (Mo^4+^ 3d_3/2_) and 236.3 eV (Mo^6+^ 3d_3/2_), attributing to the multiple chemical states of Mo induced by Fe/N doping in the MoS_2_ lattice.^[^ ^46^ ^]^ A positive binding energy shift (Δ = 0.28 eV) indicated decreased electron density around Mo, confirming p‐type doping behavior.^[^ ^47^ ^]^ The S 2p spectrum showed an increased S‐O/S^6+^ content attributed to chemical interactions between S species and FeNC. A distinct negative binding energy shift (Δ = 0.3 eV) was characteristic of S_vacs_ formation (Figure 4d). The S/Mo ratio decreased from 1.96 (pristine MoS_2_) to 1.46 (FeNC/MoS_2_‐1:2), further confirming significantly enhanced S_vacs_ generation, consistent with previously reported Fe‐induced S_vacs_ formation in the MoS_2_.^[^ ^48^ ^]^ The Fe 2p spectrum (Figure S19b, Supporting Information) exhibited enhanced Fe^2^⁺ 2p_3_/_2_ (712.1 eV) and new Fe^3^⁺ 2p_1_/_2_ (728.5 eV) signals, indicating FeS_2_ formation and Fe substitution at Mo sites.^[^ ^49^ ^]^ Further analysis of the work function through low‐binding energy measurements,^[^ ^50^ ^]^ reveals a 0.17 eV increase for FeNC/MoS_2_‐1:2 compared to MoS_2_, which was characteristic of surface electron depletion, verifying successful p‐type doping (Figures S20 and S21, Supporting Information). A detailed discussion of electron transfer is provided in the Supporting Information.

The X‐ray diffraction (XRD) tests confirmed the high crystallinity of synthesized MoS_2_, with characteristic diffraction peaks corresponding to the hexagonal 2H‐MoS_2_ phase (Figure 4e). For FeNC, the prominent peaks at ≈22° and 44° were assigned to the (002) and (100) planes of turbostratic carbon, respectively. The enhanced intensity of (002) peak was attributed to the Fe‐catalyzed graphitization process. For FeNC/MoS_2_‐1:2, coexisting MoS_2_ and FeNC peaks were observed. As the MoS_2_ content increased, the diffraction peak at 22° gradually shifted to lower angles, attributed to the formation of longer S─C bonds. The characteristic MoS_2_ peak near 58° gradually shifts to higher angles, due to the substitution of S by N incorporated into the MoS_2_ lattice. The smaller atomic radius of N (0.80 Å) compared to S (1.04 Å) induced lattice contraction. Raman spectroscopy further supported the XRD results (Figure 4f). The vibrational modes observed at 100–500 cm^−1^ corresponded to the characteristic E_1g_ (282 cm^−1^), E_2g_ (373 cm^−1^), and A_1g_ (402 cm^−1^) modes of MoS_2_.^[^ ^51^ ^]^ Pristine FeNC and FeNC/MoS_2_ composites exhibited dominant D (1350 cm^−1^, A_1g_ symmetry) and G (1580 cm^−1^, E_2g_ symmetry) bands, assigned to the carbon matrix. The intensity ratio of D to G bands (I_d_/I_g_, denoted as R‐value) indicated defect density in carbon materials.^[^ ^52^ ^]^ Pristine FeNC exhibited a low R‐value of 0.34 and a sharp 2D peak (second‐order zone boundary phonons), indicating the presence of high‐quality, multi‐layered graphene‐like structures.^[^ ^30^ ^]^ The R‐value increased with higher MoS_2_ content, indicating formation of more defects and impurities in the FeNC matrix. Meanwhile, the decreased 2D peak intensity was ascribed to thicker MoS_2_ layers, which could interfere with FeNC signal detection.

Surface energy theory provided insights into the adsorption relationships between reactive materials and gas molecules. The calculations derived from contact angle measurements (Figure 4g) revealed that MoS_2_ growth markedly enhanced both the density and proportion of dispersive active sites of FeNC (Figure 4h). Additionally, surface energy increased progressively with MoS_2_ content. These behaviors were considered beneficial for CO_2_ adsorption (details in Supporting Information).^[^ ^53^ ^]^ Figure 4i illustrated the physical interaction mechanism between FeNC/MoS_2_ and CO_2_. As a weakly reductive gas, CO_2_ underwent partial ionization in contact with S_vacs_, extracting electrons from CO_2_ and altering the charge carrier concentration. This process resulted in increased resistance for p‐type FeNC/MoS_2_, whereas n‐type composites exhibited the opposite change. The chemical reaction mechanisms were demonstrated in Figure 4j. In air, O_2_ molecule was adsorbed onto the sensing layer and captured electrons in the conduction band of the materials to form oxygen anions (O^−^, O_2_^−^, and O^2^^−^). This process generated electron depletion layers for n‐type and hole‐rich layers for p‐type (Figure S22, Supporting Information), resulting in decreased resistance in p‐type semiconductors while increasing resistance in n‐type semiconductors. Upon exposure to target gases, the adsorbed oxygen anions reacted with gas molecules, released electrons back into the conduction band, reduced depletion layer thickness, and restored resistance. In p‐type FeNC/MoS_2_, internal electron transfer rendered the MoS_2_ surface electron‐deficient, thereby suppressing oxygen ionization. The resulting lower concentration of surface oxygen ions inhibited chemical reactions. Moreover, the inherently weak oxygen ionization effect at R.T. further limited the reaction. This dual suppression effect endowed p‐type FeNC/MoS_2_ with superior CO_2_ selectivity.

**Figure 5:**
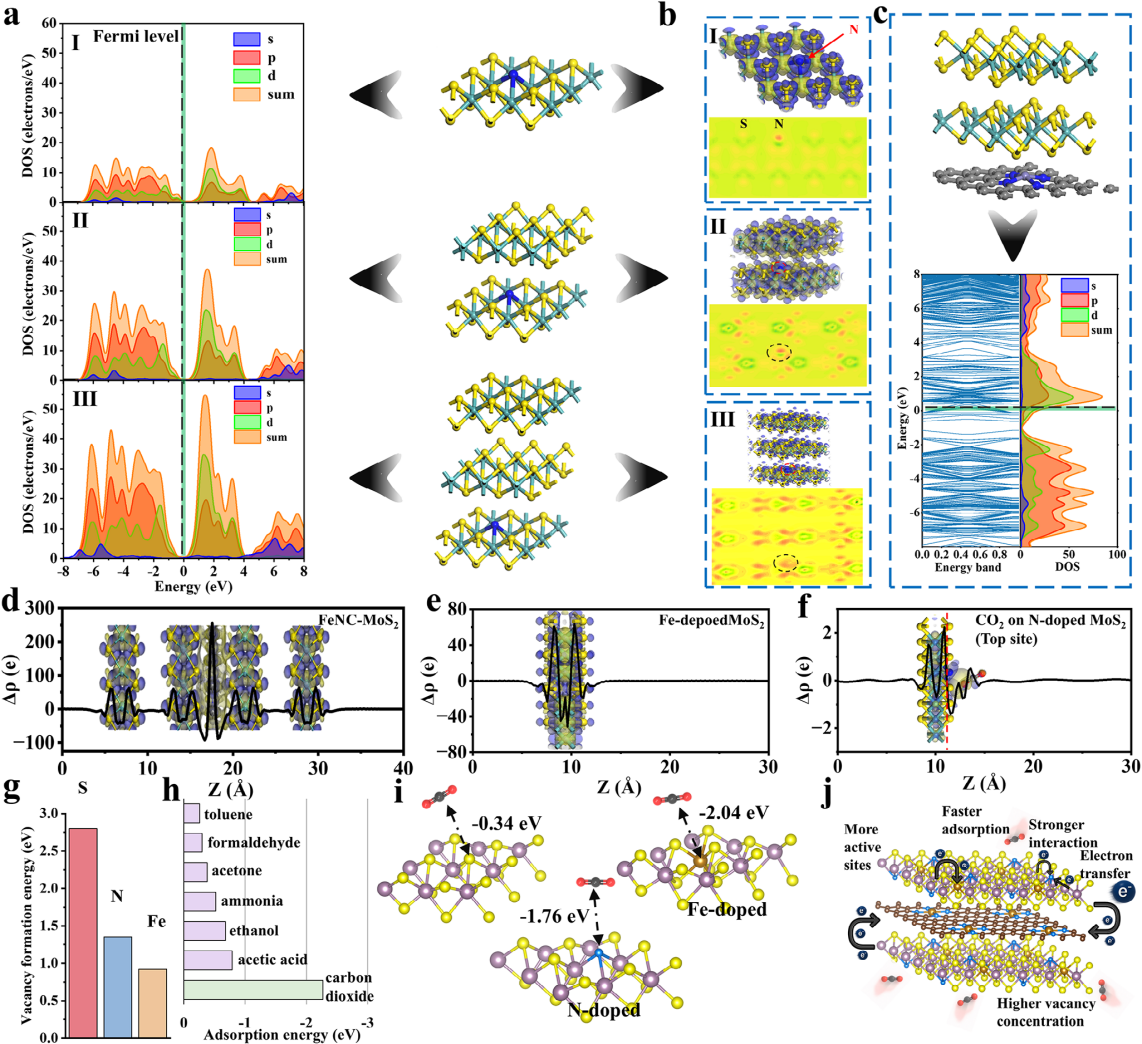
DFT calculation results of a) DOS and PDOS of the different layer numbers of N‐doped MoS_2_, b) differential charge, and c) FeNC/MoS_2_ without N doping. Statistics of differential charge along the z‐axis of d) FeNC/MoS_2_ without N doping, e) Fe‐doped MoS_2_, and f) CO_2_ on N‐doped MoS_2_, the blue is electron accumulation, yellow is electron depletion. Calculation results of g) vacancy formation energy, h) adsorption energy of different gases on N‐doped MoS_2_, and i) adsorption energy of CO_2_. j) Sensing mechanism schematic of p‐type FeNC/MoS_2_.

**Figure** **5** presented the results of DFT calculations. The density of states (DOS) analysis revealed that N‐doped MoS_2_ exhibited distinct p‐type semiconductor characteristics (Figure 5a). As the content of MoS_2_ increased, the Fermi level gradually shifted toward the conduction band, indicating a transition to n‐type semiconductor behavior. The differential charge density plots demonstrated significant charge accumulation around the N atoms (Figure 5b), confirming the effectiveness of N doping. Figure 5c showed that in FeNC/MoS_2_ without N doping, the Fermi level was located within the conduction band, which was attributed to the electron‐rich nature of pristine FeNC. The partial density of states (PDOS) analysis indicated strong orbital overlap between the s‐ and p‐orbitals of CO_2_ and doped MoS_2_, indicating favorable hybridization. Compared with single‐element doping, Fe/N co‐doped MoS_2_ exhibited a larger, broader, and more continuous DOS near the Fermi level upon CO_2_ adsorption. Strong hybridization occurred between the p states of C and O in CO_2_ and the N 2p orbitals, and the d states of Fe/Mo, resulting in pronounced electronic coupling (Figure S23, Supporting Information). Further statistical analysis of charge distribution along the z‐axis revealed substantial electron accumulation at the FeNC region in FeNC/MoS_2_ (Figure 5d). This electron transfer exceeded the typical range of *van der Waals* interactions, indicating the formation of chemical bonds between FeNC and MoS_2_, consistent with the XPS results. Figure 5e showed that Fe doping induced charge redistribution in MoS_2_, causing pronounced charge accumulation around the Fe sites compared to pristine MoS_2_ (Figure S24a, Supporting Information). Analysis of the spatial charge distribution after CO_2_ adsorption revealed charge transfer between CO_2_ and MoS_2_, with the variation magnitude consistent with physisorption (Figure S24b, Supporting Information). Examination of N‐doped configurations (top and bottom positions) demonstrated that N doping enhanced charge transfer between CO_2_ and MoS_2_, with stronger interactions at the top site (Figure 5f; Figure S24c, Supporting Information). This significant charge transfer is also observed in the Fe and N co‐doped system (Figure S24d‐f, Supporting Information). Figure 5g presented the calculated vacancy formation energies. The Fe‐doped system exhibited the lowest energy barrier for S_vacs_ formation, strongly supporting the validity of the proposed design strategy (computational models shown in Figure S25, Supporting Information). As shown in Figure 5h, the N/Fe‐doped S_vacs_‐MoS_2_ model displayed higher CO_2_ adsorption energy than interfering gases. Furthermore, even in the absence of vacancies, the presence of N and Fe dopants enhanced CO_2_ adsorption by modulating the local electron density (Figure 5i). A more detailed discussion can be found in the Supporting Information. The complete statistical results were summarized in **Table** **2**, Tables S1 and S2 (Supporting Information), with the corresponding computational models shown in Figures S26 and S27 (detailed discussion in Supporting Information).

**Table 2:** Calculation results of adsorption energy and adsorption heat.

Models	E_ads_ [eV]	D_ads_ [Å]	Adsorption heat [Avg, kJ/mol]
Pure MoS_2_	−0.34	3.76	8.03
N‐MoS_2_	−1.76	3.18	8.43
3N‐MoS_2_	−1.63	3.23	9.28
3N, Fe doped MoS_2_	−1.78	2.83	9.37
S_vacs_‐MoS_2_	−2.02	3.04	7.51
N doped S_vacs_‐MoS_2_	−2.15	2.96	7.64
N, Fe doped S_vacs_‐MoS_2_	−2.27	2.86	7.65
N, Fe doped S_vacs_‐FeNC/MoS_2_ (Bottom site)	−2.52	3.27	16.72
N, Fe doped S_vacs_‐FeNC/MoS_2_ (TOP site)	−2.63	2.44	16.86

The results demonstrated that Fe, N, and S_vacs_ all significantly enhanced CO_2_ adsorption, with this enhancement being equally pronounced for both top and bottom sites. Moreover, no strict linear correlation was observed between the most stable adsorption distance and the adsorption energy, a phenomenon commonly reported for physisorption processes dominated by *van der Waals* interactions.^[^ ^54^ ^]^ The adsorption heats calculated via grant canonical Monte Carlo (GCMC) were generally consistent with the trends observed in the adsorption energies, with values falling in the range characteristic of physisorption. The p‐type FeNC/MoS_2_ model exhibited both higher adsorption energy and heat compared to other models, which was attributed to the presence of vacancies and its superior internal electronic regulation, collectively enhancing CO_2_ adsorption (Figure 5j).

**Figure 6:**
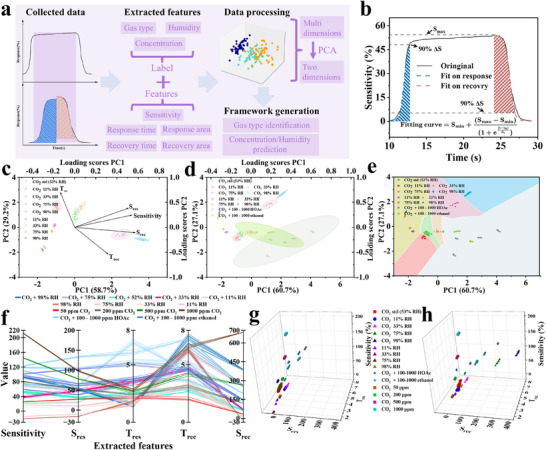
a) Schematic of the data extraction and processing, b) feature extraction from the response curves. PCA scatter plot and confidence interval of extracted features c) CO_2_, CO_2_ + RH, RH, and d) CO_2_, CO_2_ + RH, CO_2_ + interfering gases, RH, and e) decision map of seven different groups. f) Parallel coordinate plot of all extracted features. 3D scatter plot of g) S_res_ and T_res_, and h) S_rec_ and T_rec_. The standard condition (std): 100 ppm CO_2_, 53% RH and 25 °C.

A ML strategy was developed to recognize and predict gas compositions in mixed environments using a single sensor (based on FeNC/MoS_2_‐1:2) (**Figure** **6**a). Sensor response curves were collected under different conditions, including humidity levels, gas concentrations, and interfering gases. Each response curve was considered a combination of a gas adsorption (response) phase and a gas desorption (recovery) phase. Therefore, the response and recovery curves can be individually fitted using exponential growth and decay functions, respectively. A gas transport mechanism based on Boltzmann statistics and transport theory was applied to fit the curves, as defined in Equation (1):

(1) $$Fittingcurve=S_{min}+\frac{S_{max}-S_{min}}{1+e^{\frac{t-t_{0}}{d_{t}}}}$$

where S_min_, S_max_, t_0_, t and d_t_ were the minimum sensitivity, maximum sensitivity, initial time, ending time, and time integration values within the fitting range, respectively.^[^ ^55^ ^]^ Subsequently, five characteristic parameters were extracted: sensitivity value, response time (T_res_), response area (S_res_), recovery time (T_rec_), and recovery area (S_rec_) (Figure 6b). These features were subjected to principal component analysis (PCA) for dimensionality reduction, projecting the multi‐dimensional feature dataset into a 2D principal component (PC) space. A supervised ML strategy was employed, using the dimensionally reduced PC data as feature inputs to train a regression surface for predictive functionality. This was a visualized ML approach, enabling the projection of discrete regression‐derived 3D surfaces onto a 2D classification map within the PCA space.

A parallel coordinate plot of the five characteristic parameters from the response curves of humid air and CO_2_ in humid air was shown in Figure S28 (Supporting Information), illustrating clear clustering and providing intuitive insights into the data distribution. Figure 6c presented the PCA results, showing the distribution of parameters including the variance of two principal components (PC1 and PC2) and the loading scores of the five extracted features. No overlap was observed among scatter points representing different categories, with distinct confidence intervals confirming the effectiveness of the PCA‐based processing. As the dataset was expanded to include CO_2_ response curves under varying concentrations of interfering gases, the parallel coordinate plot became increasingly complex (Figure 6d; Figure S29, Supporting Information). Despite some overlapping in confidence intervals, scatter points maintained distinct distribution patterns, indicating discernible group differences within the PC space. For classification, the dataset was categorized into seven classes and assessed multiple algorithms, including random forest (RF), support vector machine (SVM), k‐nearest neighbor (KNN), and long short‐term memory (LSTM) algorithms to identify the optimal model. The decision boundaries generated by the KNN algorithm (K = 5) were shown in Figure 6e, providing distinct and well‐separated classifications for CO_2_ under standard test conditions (green), pure humidity (yellow), CO_2_ at different RH (11%, 33%, 75%, 98% RH; red, brown, pink, blue), and CO_2_ in the presence of various interfering gases (gray). The confusion matrix showed a 97.14% recognition accuracy for CO_2_, confirming reliable detection under varying RH and interfering gases using a single sensor (Figure S30, Supporting Information). Additional characteristic values from response curves at different CO_2_ concentrations were added, and the resulting parallel coordinate plot is shown in Figure 6f. The corresponding parameters were visualized in 3D space, where distinct clusters demonstrated that our classification strategy remains efficient (Figure 6g,h; Figures S31 and S32, Supporting Information).

**Figure 7:**
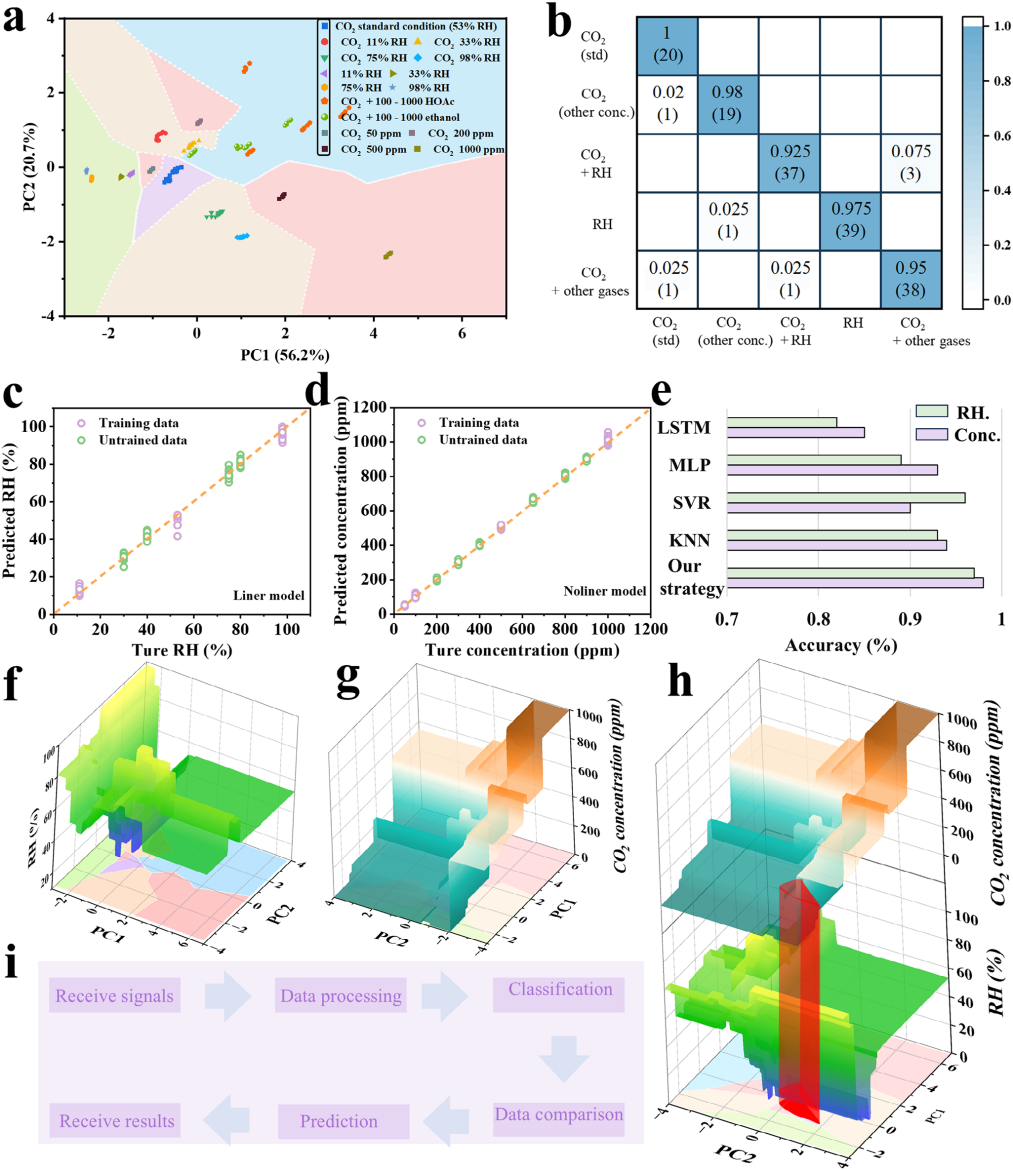
a) Decision map of five different groups, b) confusion matrix for five different groups. Predicted concentrations versus true concentrations for c) RH and d) CO_2_. e) Prediction accuracy of our strategy and other ML models. Regression surface of the predicted concentrations in PC spaces for f) RH and g) CO_2_, and h) stacked plots of (f) and (g). i) Data processing schematic of the sensor for classification and prediction.

The adopted classification strategy was illustrated in **Figure** **7**a, comprising five categories: CO_2_ under standard test conditions (purple), pure humidity (green), CO_2_ with various interfering gases (blue), CO_2_ at different RH (beige), and different concentrations of CO_2_ (red). The KNN algorithm remained effective, clearly distinguishing even in densely populated data regions. The confusion matrix confirmed the sensor's ability to differentiate CO_2_ concentrations with a high recognition accuracy of 95.6% (Figure 7b). To predict gas concentration and humidity, regression models from the scikit‐learn library were applied. Based on prior experimental results (Figure 3k), two separate models were trained: a nonlinear model for CO_2_ and a linear model for humidity. Due to strong nonlinear characteristics within the dataset, a hybrid learning approach was adopted to enhance model performance. Specifically, a non‐parametric RF regression model was trained on the KNN‐generated decision boundaries to produce regression surfaces. Only limited dataset was used for training, including 11% RH and 98% RH for humidity, and 50, 100, and 1000 ppm for CO_2_, excluding other intermediate concentrations. The prediction results were shown in Figure 7c,d, where purple dots represented training data and green dots represented untraining data. Model accuracy (R^2^) was indicated by calculating the residuals between predicted and true values, as defined in Equation (2):

(2) $$Accuracy\;\;\left(R^{2},\%\right)=1-\frac{\sum {\left(y-y_{i}\right)}^{2}}{\sum {\left(y-{\bar{y}}_{i}\right)}^{2}}$$

where *y* was truth value, *y_i_* was the predicted value, ${\bar{y}}_{i}$ was the mean of the true values, and $y-{\bar{y}}_{i}$ was the total variance. The results showed that R^2^ values (red dashed line) reached 97% and 98% for humidity and CO_2_ concentration, respectively, significantly higher than other models (Figure 7e). Regression surfaces for RH and CO_2_ concentration were generated in the PC space at predefined discrete resolution levels (Figure 7f,g). Figure 7h presented a stacked plot of classification results, showing the projection of predicted concentration regression surfaces onto the 2D PC space overlaid with the original classifier's decision boundaries. Specifically, the detected signals were processed and projected onto their corresponding regions of the CO_2_ and RH regression surfaces (highlighted as red bands). Upon validation, data points that were not included in the regression model's training set produced predictions closely matching actual measured values. The complete data processing and projection workflow was illustrated in Figure 7i. These results demonstrated that the proposed hybrid learning strategy can effectively manage datasets with nonlinear discriminative features, establishing a visualized ML framework. Moreover, this strategy exhibited strong potential for extension to broader sensing applications.

**Figure 8:**
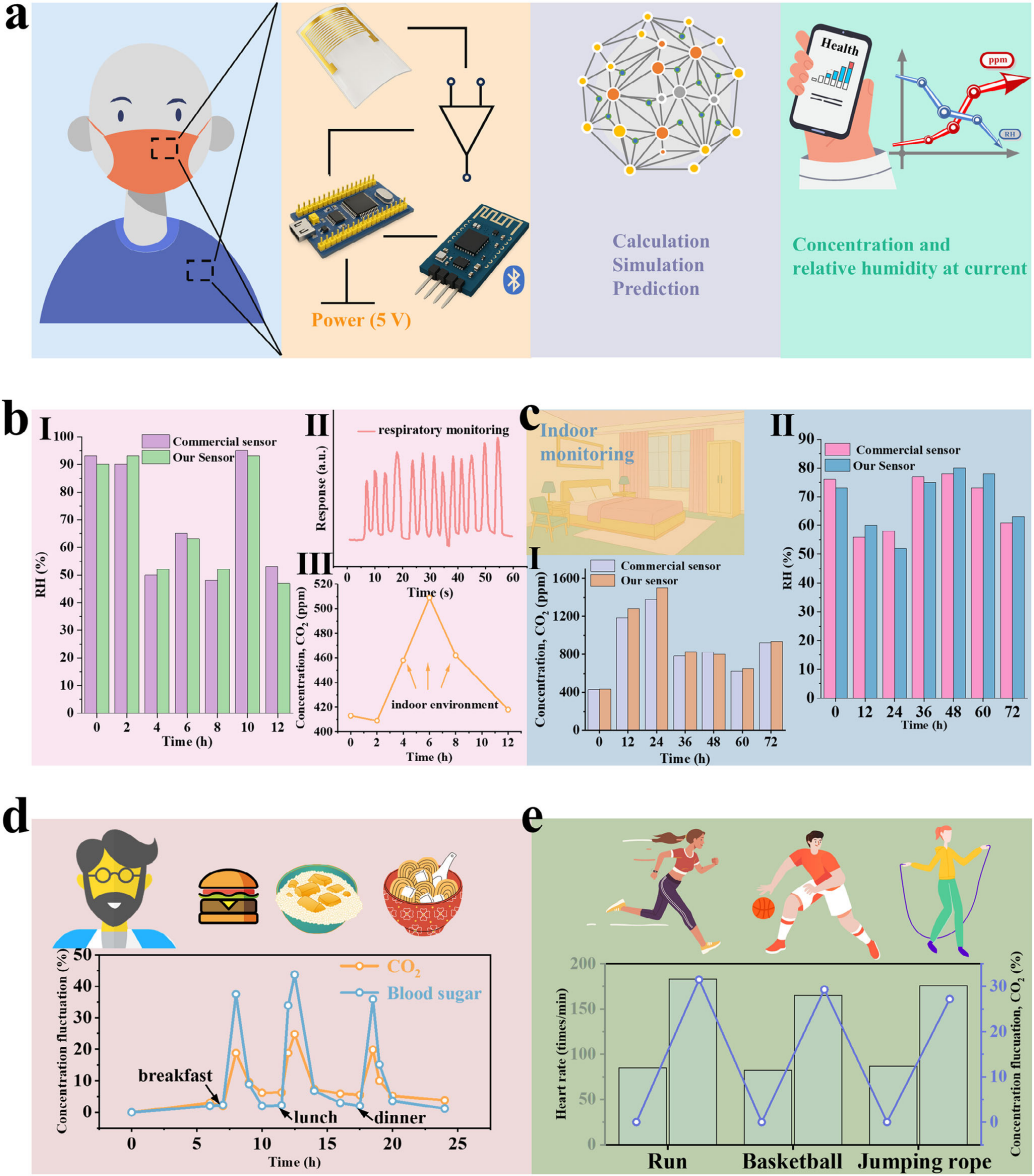
a) Schematic showing the overall signal processing flow and smart portable sensor components. Our portable sensor was assessed under various conditions and benchmarked against commercial ones. b) I) RH, II) respiratory monitoring, III) outdoor and indoor CO_2_ concentrations. c) Indoor monitoring, I) CO_2_, II) RH. d) Relationship between CO_2_ and blood glucose values. e) Relationship between CO_2_ and heart rates.

To evaluate the feasibility of the developed smart sensor (S‐Sensor) in real environments, a portable device was fabricated (**Figure** **8**a). Owing to the R.T. operating properties of the sensing material, satisfying the requirements for miniaturized and portable devices. As a result, the S‐Sensor can be seamlessly embedded into masks, clothing, or electronic devices, providing users with real‐time CO_2_ and humidity data via smartphones or smartwatches for continuous monitoring. Figure 8b presented the monitoring performance of the S‐Sensor under different environmental conditions. The CO_2_ concentration readings from the S‐Sensor showed strong agreement with the commercial sensor (I). Furthermore, the sensor was integrated into a face mask to monitor respiration in real time (II). Due to the S‐Sensor was embedded between two layers of nonwoven fabric within the mask, making it difficult to obtain accurate CO_2_ and RH values, the relative trends remained clearly detectable. Simultaneous prediction of RH was also achieved (III). Figure 8c presented indoor real‐time monitoring results, where both CO_2_ concentration (I) and RH prediction values (II) closely matched commercial devices. To explore more attractive applications, exhaled breath samples were collected from a subject before and after food intake, with CO_2_ concentrations analyzed after dilution (details in Supporting Information). CO_2_ levels were compared with blood glucose values measured by a non‐invasive glucose‐monitoring smartwatch, revealing a correlation between exhaled CO_2_ concentration and blood glucose levels (Figure 8d). Similarly, exhaled breath samples collected after different physical activities were compared with heart rate data from a smartwatch, demonstrating a strong correlation between CO_2_ levels and heart rate (Figure 8e). The PCA–regression model was validated by held‐out tests, cross‐validation, and field comparisons with a commercial sensor, confirming its robustness and ability to generalize beyond the training conditions. These findings highlighted not only the excellent sensitivity and selectivity of the FeNC/MoS_2_ material and the effectiveness of the ML framework but also the significant potential of the S‐Sensor for non‐invasive health and medical monitoring applications.

## 3 Conclusion

In summary, a chemically tunable strategy was established for synthesis of FeNC/MoS_2_ with switchable n‐type and p‐type characteristics for smart sensor applications. FeNC/MoS_2_ was prepared via decoration of N/Fe‐rich carbon nanosheets, with MoS_2_ through high‐temperature pyrolysis of (NH_4_)_2_MoS_4_, a process allowing the MoS_2_ phase to form on FeNC surface and also the dopant elements to migrate into the MoS_2_ lattice for p‐type doping. The growth thickness of the MoS_2_ thus the morphology of FeNC/MoS_2_ was controllable by the adding amount of Mo precursor. Due to the limited concentration of p‐type dopants, as the MoS_2_ content increased, the dopants could not migrate into the more distant MoS_2_ layers, which prevented the newly formed n‐type MoS_2_ from converting to p‐type, thus the composites exhibited n‐type characteristics. The p‐type FeNC/MoS_2_‐1:2 exhibited excellent sensitivity and selectivity toward CO_2_, achieving a stable and repeatable detection limit as low as 50 ppm at R.T., owing to a synergistic modulation mechanism within the composite. Specifically, Fe promoted the formation of abundant vacancies in MoS_2_, while N functioned as the primary dopant, substituting S to induce p‐type behavior. The differences in work function and electronegativity facilitated electron transfer from MoS_2_ to the carbon nanosheets, with electron density further localized around the N and Fe sites within MoS_2_. This synergistic effect enhanced CO_2_ adsorption by increasing vacancy concentration and active site density, while simultaneously suppressing oxygen ionization on the material surface. These insights were strongly supported by DFT and GCMC simulations. A machine learning model was subsequently developed based on a single sensor, capable of accurately classifying and predicting gas concentrations under interfering conditions. This hybrid ML framework effectively captured nonlinear response features and achieved accuracy exceeding 95%. Moreover, the model demonstrated high reliability in predicting humidity and CO_2_ concentrations beyond the training dataset. An intelligent sensor was fabricated, which not only detected environmental variations but also captured and predicted CO_2_ fluctuations in human breath across different physiological states. Correlating the sensor outputs with physiological data revealed strong associations, highlighting the promise of this CO_2_ sensor for applications in noninvasive health monitoring and medical diagnostics.

## 4 Experimental Section

### Synthesis of FeNC Nanosheets

0.2 g of dopamine hydrochloride (PDA) and 2 g of FeCl_3_·6H_2_O at a 1:10 mass ratio were dissolved in 2 and 5 mL of deionized water (DI), respectively. Sequentially, the solutions were mixed and stirred continuously for 1 h. After aging for 24 h, the solution was transformed into a viscous dark liquid (Figure S1, Supporting Information). The complex solution was then freeze‐dried and then transferred into a furnace under argon (Ar) atmosphere at 800 °C (5 °C min^−1^) for 2 h. The obtained product was treated with 1 M sulfuric acid (H_2_SO_4_) for 12 h to remove iron oxide. The product was collected by filtration and repeatedly washed to neutrality, then dried in an oven at 60 °C for 12 h to yield the 2D Fe and N rich carbon nanosheets.

### Growth of MoS_2_ on FeNC Nanosheets

FeNC nanosheets and ammonium tetrathiomolybdate ((NH_4_)_2_MoS_4_) were mixed at different mass ratios (1:0.5, 1:1, 1:2, 1:4, 1:5. 1:5.5, 1:6, 1:8, and 1:10) by grinding. Then, the mixture was treated at a pyrolysis temperature of 600 °C for 2 h under H_2_/Ar (5% H_2_), and (NH_4_)_2_MoS_4_ thermally decomposed and grew MoS_2_ onto the nanosheet surface. Bulk MoS_2_ was obtained by direct pyrolysis of (NH_4_)_2_MoS_4_. The resulting samples were denoted as FeNC/MoS_2_‐x:x, where x corresponded to the precursor mass ratio of FeNC to (NH_4_)_2_MoS_4_ (e.g., FeNC/MoS_2_‐1:2 represented a 1:2 precursor mass ratio).

### Gas Measurements

Gas sensors were measured using a gas sensing characterization system (Beijing Zhong‐ke Tech Co.). A voltage of 5 V was used to evaluate the sensor resistance, and the relative humidity (RH) was controlled by different saturated salt solutions. The concentration of CO_2_ was carefully controlled by flowmeters. To simulate real‐world conditions, the CO_2_ concentration in this work was adjusted based on the atmospheric environment as the primary reference. The atmospheric environment was defined as 53% RH and 400 ppm CO_2_, and the baseline resistance of all sensors in this work was obtained under these conditions. For example, the sensors were first placed in the atmospheric environment, and then a certain amount of CO_2_ was introduced to increase the ambient concentration by 100 ppm to obtain the response curve of 100 ppm. The concentration of volatile organic compounds (VOCs) was manipulated by using the static gas distribution method. Preliminary tests were conducted before selecting interfering gases. Gases that showed little‐to‐no response at R.T. (e.g., methane) were excluded from further consideration, and several common VOCs were then selected. By injecting a predetermined volume of the target solution into the 20 L chamber using a micro syringe at R.T. (25 °C), the solution evaporated, resulting in the desired gas concentration. It should be noted that the gases were prepared in the ambient air, meaning that the VOCs also contained water vapor (= air humidity). To ensure the accuracy of the results, the experiments were repeated five times, and the average values were calculated. The sensitivity is calculated as Equation (3):

(3) $$Sensitivity\left(\%\right)=\frac{R_{\mathrm{gas}}-R_{\mathrm{air}}}{R_{\mathrm{air}}}\times 100$$

where R_gas_ and R_air_ were the resistance of sensor exposed to the target gases and air condition, respectively. The response and recovery times were calculated from the transient response as the time taken for 90% of the total resistance change in the response/recovery profile by the sensor.

### Calculation Details

The plane‐wave‐based DFT method with Perdew–Burke–Ernzerhof (PBE) of generalized gradient approximation (GGA) exchange‐correlation functional as implemented in the Vienna ab initio simulation package (VASP).^[^ ^56^, ^57^ ^]^ A plane‐wave energy cutoff of 500 eV was used. The total energy convergence criterion was set to 1 × 10^−^⁵ eV, while the force convergence threshold for ionic relaxation was 0.02 eV Å^−1^. Brillouin zone integrations were performed using a Gaussian smearing of 0.1 eV. The van der Waals interaction was treated with the DFT‐D3 method with Becke–Johnson damping.^[^ ^58^ ^]^ The supercells of 4 × 4 × 1 and 3 × 3 × 1 were chosen as the research models. For the Brillouin zone sampling, Monkhorst–Pack k‐point meshes of 3 × 3 × 1 and 5 × 5 × 1 were used for the 4 × 4 × 1 and 3 × 3 × 1 supercells, respectively, which were tested to be sufficient for total energy convergence. To avoid spurious interactions between periodic images in the out‐of‐plane direction, a vacuum spacing of 20 Å was applied.

The adsorption energy (E_ads_) was calculated followed by Equation (4), where E_materials + gas_ was the total energy of the system, E_materials_ was the energy of materials, and E_gas_ was the energy of target gas.

(4) $$E_{\mathrm{ads}}=E_{\mathrm{materials}\;+\;\mathrm{gas}}\;\;-\left(E_{\mathrm{materials}}+E_{\mathrm{gas}}\right)$$

The vacancy formation energy ($E_{f}^{v}$) was calculated by Equation (5), where *E*_vac_ was the energy of a unit cell containing vacancy, *E*_bulk_ was the energy of a unit cell without defects, and μ was the chemical potential of removed atoms.

(5) $$E_{f}^{v}=E_{\mathrm{vac}}\;\;-E_{\mathrm{bulk}}+\mu$$

Gas adsorption heat simulations were investigated using grand canonical Monte Carlo (GCMC) simulations. The universal force field was used to describe the energy parameters of all atoms, and the Ewald summations method was used to compute the electrostatic interactions.

### Fabrication of Portable Gas Sensor System

The sensing material was dispersed into ethanol and coated onto a polyethylene terephthalate (PET) based electrode. The sensing system consisted of an electrode, an amplifier, a microprocessor, and a Bluetooth module (Figure S2, Supporting Information). The module transmitted data to a smartphone or computer for collection. The power was provided by a battery with 3.6 to 6 V.

### Data Processing

To process data, curves were fitted, and features were extracted. Next, principal component analysis (PCA) was used to reduce features from multi‐dimensional to 2D for data visualization. Then, the data were classified and a decision boundary was generated for regression prediction. The ML models were sourced from the scikit‐learn ML library. Matlab, Python, Vscode, and Origin were used for signal processing, feature extraction, and data visualization.^[^ ^59^ ^]^

## Conflict of Interest

The authors declare no conflict of interest.

## Author Contributions

Y.G. performed investigation, analysis, methodology, wrote the original draft, wrote, review and edited the final manuscript. Y.W. performed data curation. J.A. performed review. G.L. performed methodology. L.W. performed methodology. Z.W. performed methodology. J.Y. performed project administration, funding acquisition, and review. Q.L. performed project administration, funding acquisition, and review.

## Supporting information



Supporting Information

## Data Availability

The data that support the findings of this study are available from the corresponding author upon reasonable request.
