# Supplementary material for: Grow p‐type MoS2 on FeNC for CO2 Sensing in Complex Environments with Intelligent Recognition
Source: Adv Sci (Weinh). 2025 Oct 24;13(1):e12595. doi: 10.1002/advs.202512595 (PMC12767046; doi:10.1002/advs.202512595)
Supplement: Supplementary file 1 — Supporting Information [file ADVS-13-e12595-s001.docx]

**Grow p-type MoS_2_ on FeNC for CO_2_ Sensing in Complex Environments with Intelligent Recognition**

Yuefeng Gua^,b^, Yuhao, Wang^a^, Jing Ai^b^, Gongjie Liu^a^, Sadaf Saeedi Garakani^c^, Lisi Wei^a^, Zeen, Wu^b^, Jiayin Yuan^b*^, Qiuhong Li^a*^

^a^ *School of Electronic Science and Engineering, Xiamen University, Xiamen, 361005, China*

^b^ *Department of Chemistry, Stockholm University, Stockholm, 10691, Sweden*

^c^ *Division of Chemical Engineering, Chalmers University of Technology, Gothenburg, 41296, Sweden*

**Corresponding Author*

Email: [Jiayin.yuan@su.se](mailto:Jiayin.yuan@su.se); [liqiuhong@xmu.edu.cn](mailto:liqiuhong@xmu.edu.cn)

**Characterization**

**Raw materials**

Ferric chloride hexahydrate (FeCl_3_·6H_2_O), Dopamine hydrochloride, ammonium tetrathiomolybdate ((NH₄)₂MoS₄), ethanol, acetone, ammonia, acetic acid, formaldehyde, and toluene were purchased from Sigma-Aldrich. Sulfuric acid (98%) was purchased from TCI. All of the reagents were used as received without further purification.

**Apparatus**

The crystal structure of the materials was investigated by a powder X-ray diffraction (XRD) system (Rigaku Ultima IV) with Cu Ka radiation from 10 to 80° (2θ) at a scan rate of 10◦ min^−1^, and a Microconfocal Raman Spectrometer (Raman, lDSPeC ARCTlC). Field-emission scanning electron microscopy (FESEM, SUPRA-55, ZEISS, Germany) and transmission electron microscopy (TEM, F30) were used to study the morphology and microstructure. X-ray photoelectron spectroscopy (XPS) was performed by employing a Thermo-Fisher EScalab 250Xi XPS system, with Al Ka radiation.

**Surface energy test**

The surface energy properties were characterized using a contact angle measurement system (Dataphysics, OCA20). Contact angles were performed using water and diiodomethane and used Eq (1) and (2) to calculate surface energy ($\gamma_{f}$), dispersive component ($\gamma_{f}^{d}$), and polar component ($\gamma_{f}^{P}$):

$${\gamma_{l}\left( 1+cos\theta\right)=2\left( \gamma_{l}^{p}\gamma_{f}^{p} \right)}^{\frac{1}{2}}+{2\left( \gamma_{l}^{d}\gamma_{f}^{d} \right)}^{\frac{1}{2}} (1)$$

$$\gamma_{f}=\gamma_{f}^{p}+\gamma_{f}^{d} (2)$$

where the $\gamma_{l}$, $\gamma_{l}^{p}$ and $\gamma_{l}^{d}$ are the surface tension of the liquid, polar and dispersive component, respectively. Every test was repeated five times and the average value of contact angles was calculated.


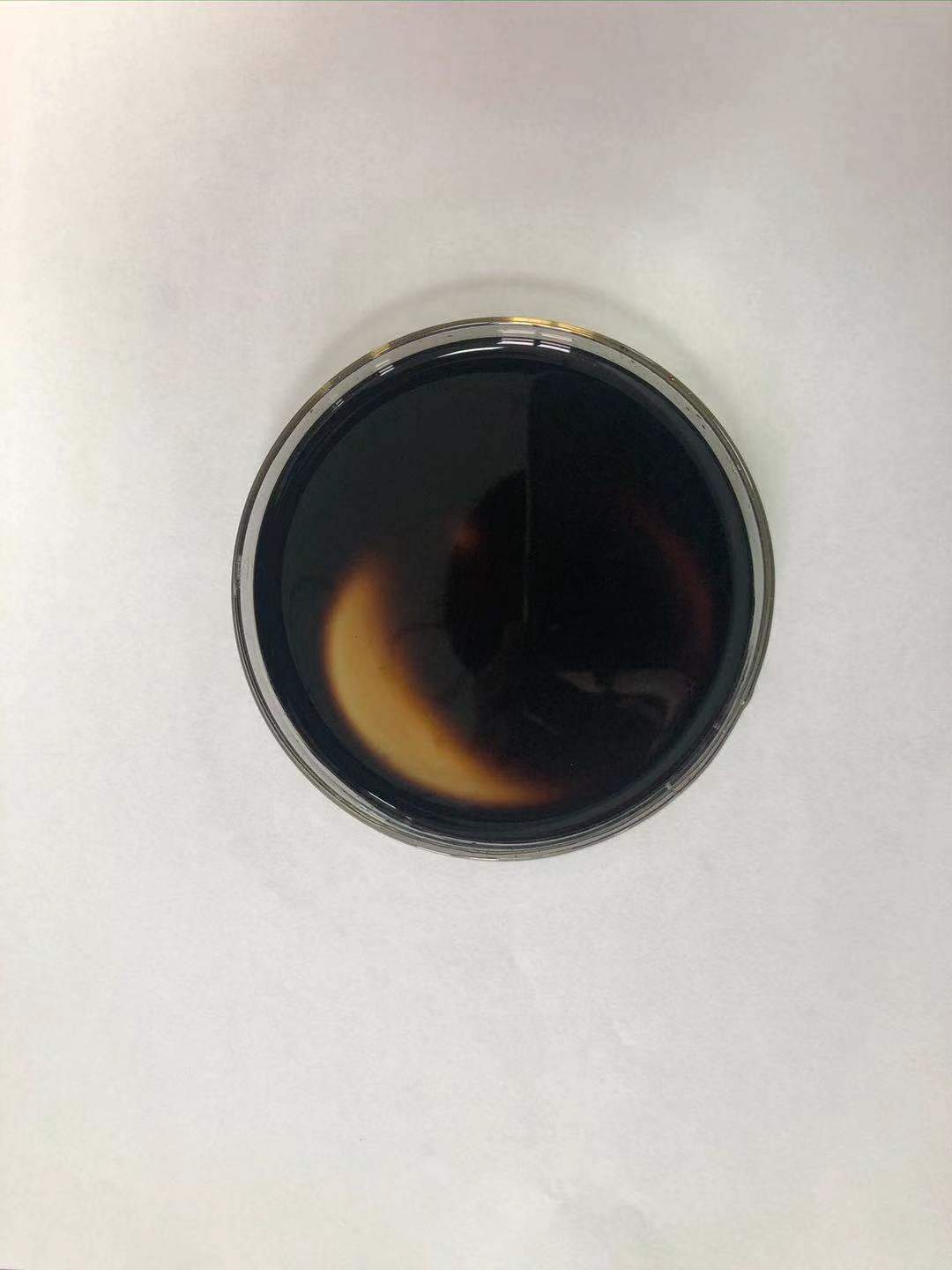


Figure S1. Photograph of dopamine hydrochloride (PDA) and FeCl_3_·6H_2_O organic-inorganic hybrid.


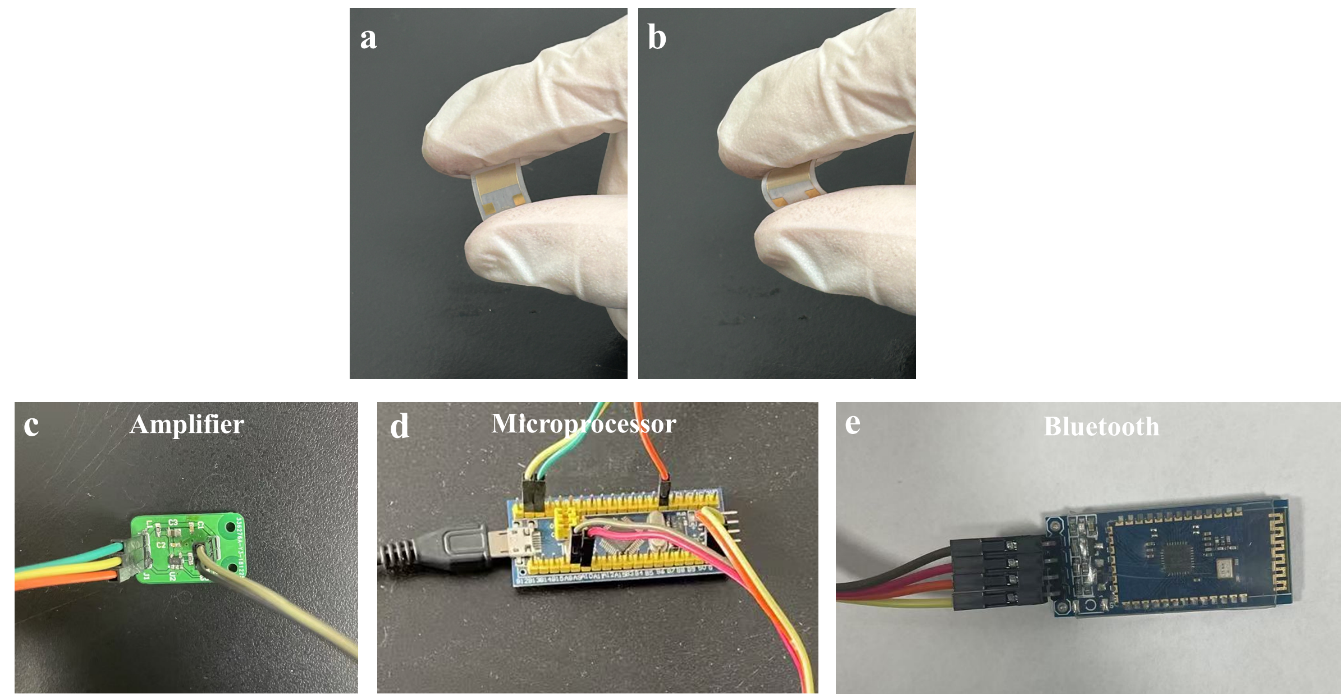


Figure S2. (a), (b) Photograph of PET electrode, (c) Amplifier, (d) Microprocessor, and (e) Bluetooth module.


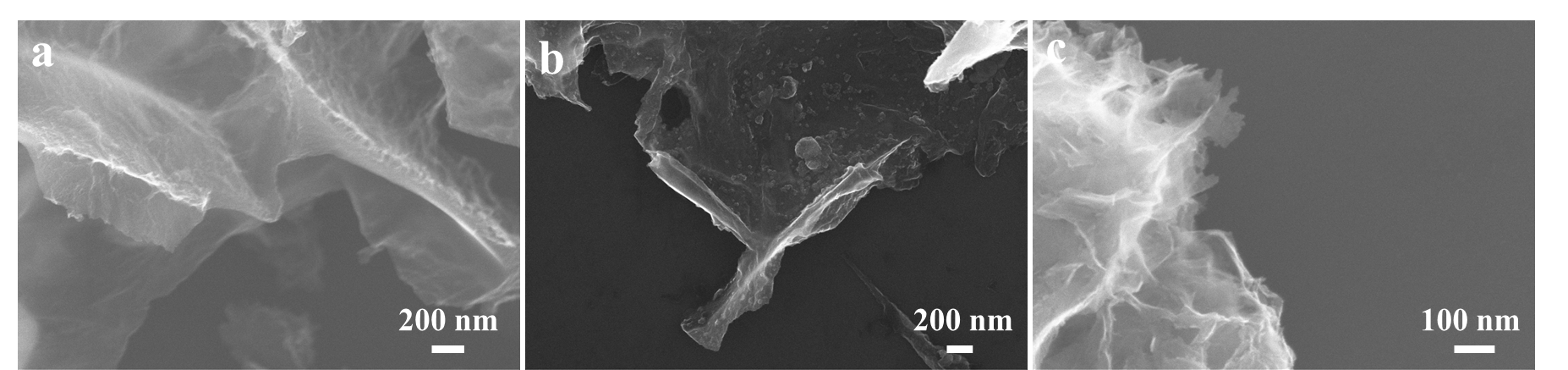


Figure S3. (a-c) SEM images of FeNC.


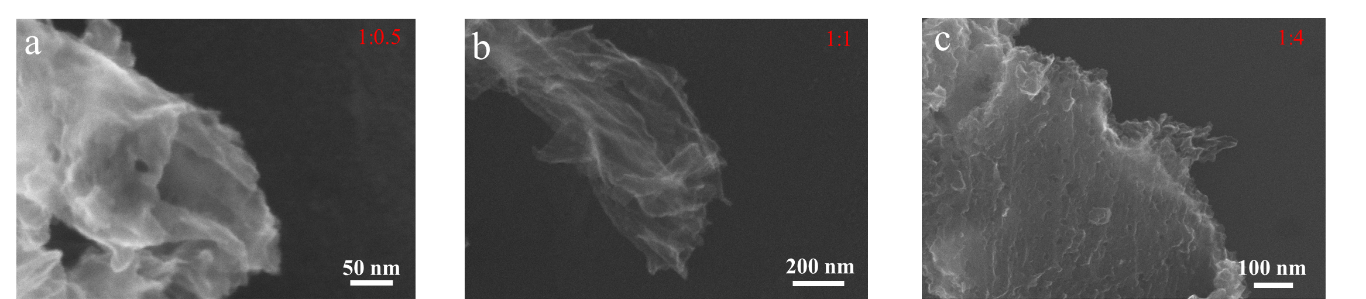


Figure S4. SEM images of (a) FeNC/MoS_2_-1:0.5 (a) FeNC/MoS_2_-1:1 and (b) FeNC/MoS_2_-1:4.


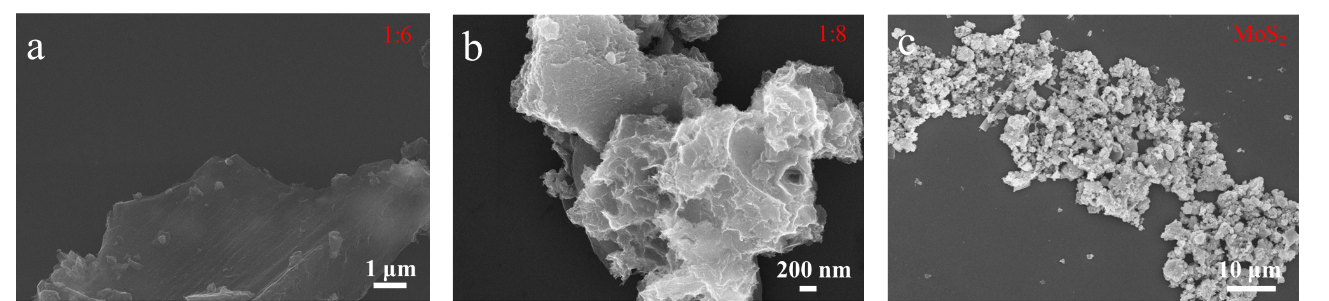


Figure S5. SEM images of (a) FeNC/MoS_2_-1:6, (b) FeNC/MoS_2_-1:8, and (c) pristine MoS_2_.


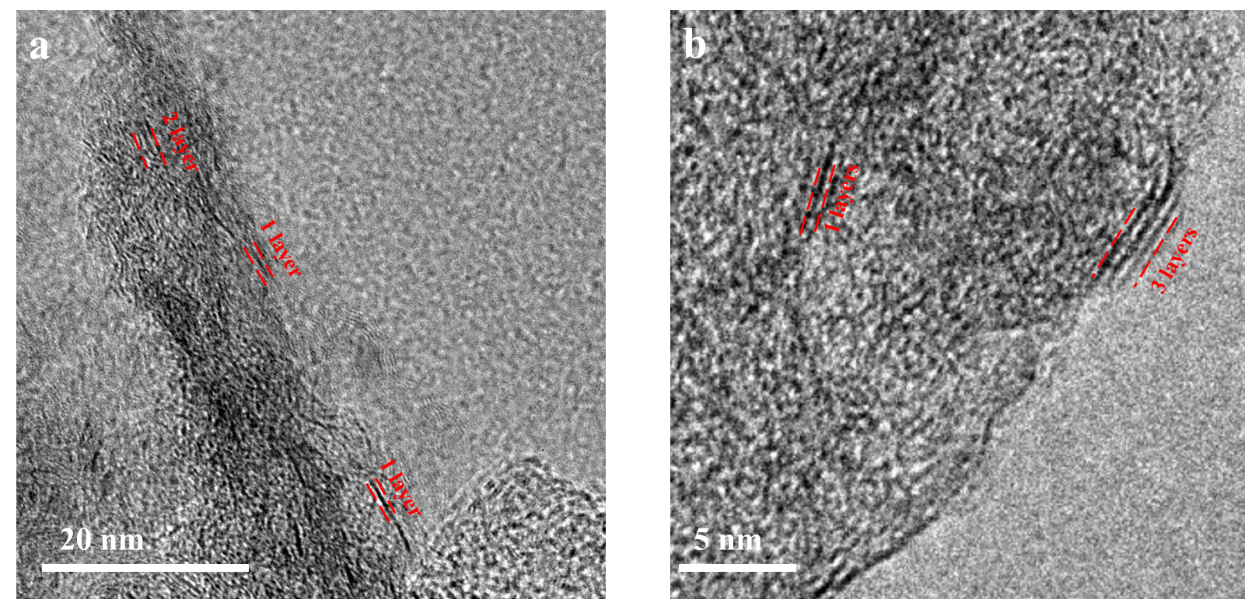


Figure S6. (a-b) TEM images of FeNC/MoS_2_-1:1.


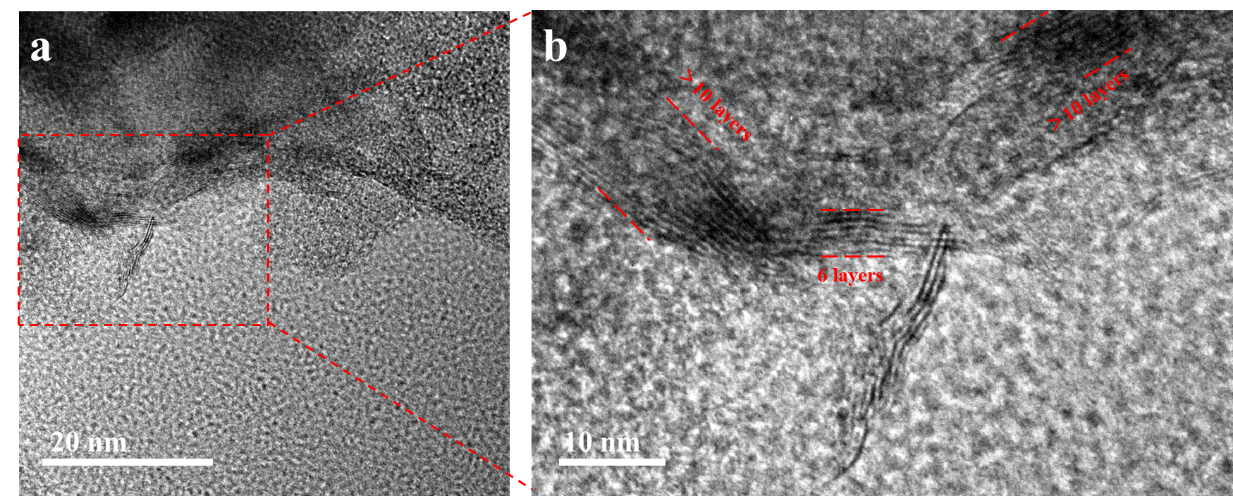


Figure S7. (a-b) TEM images of FeNC/MoS_2_-1:6.


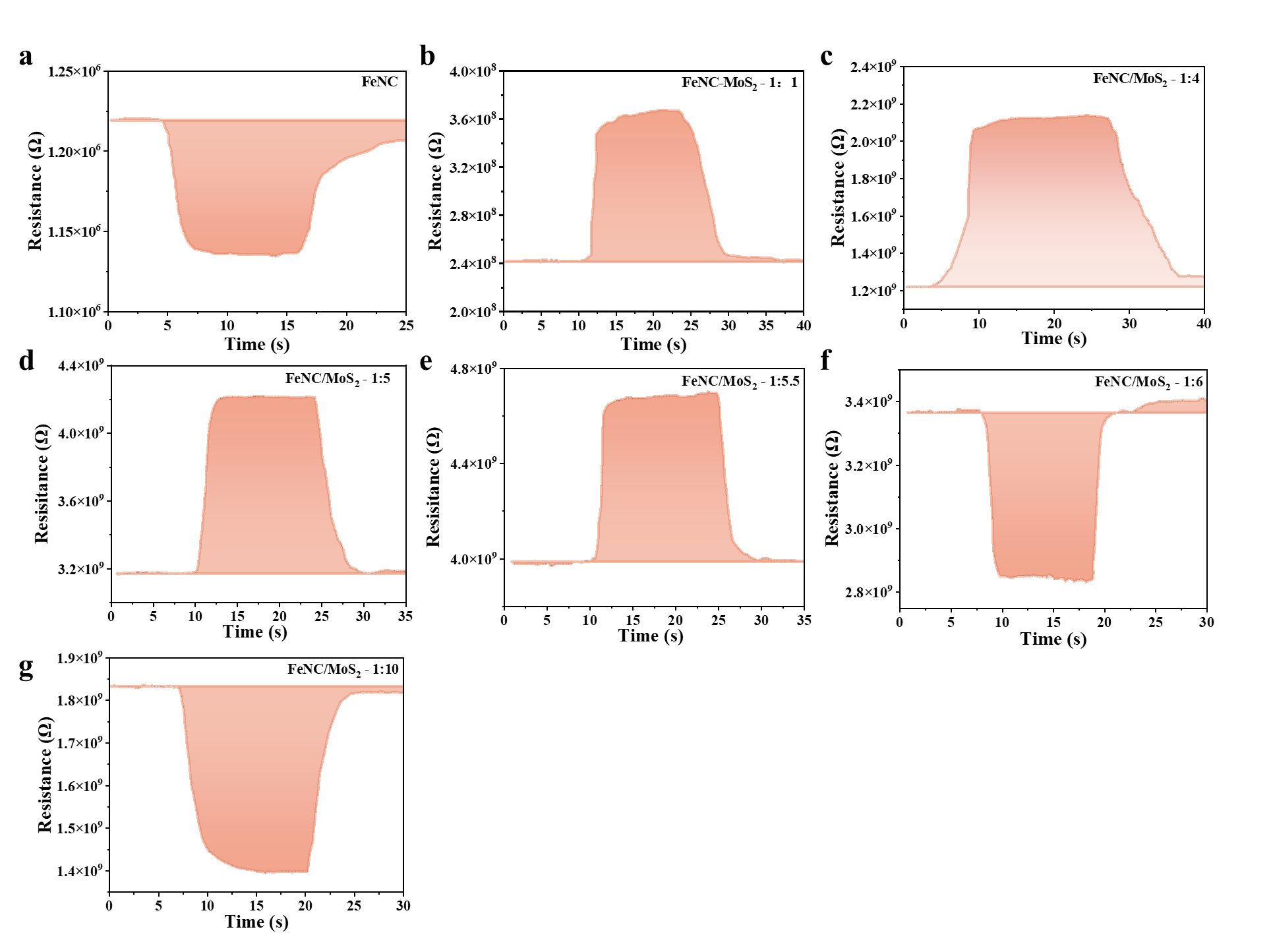


Figure S8. Response curves for 200 ppm CO_2_ of (a) FeNC, (b) FeNC/MoS_2_-1:1 (c) FeNC/MoS_2_-1:4, (d) FeNC/MoS_2_-1:5, (e) FeNC/MoS_2_-1:5.5, (f) FeNC/MoS_2_-1:6, (g) FeNC/MoS_2_-1:10.


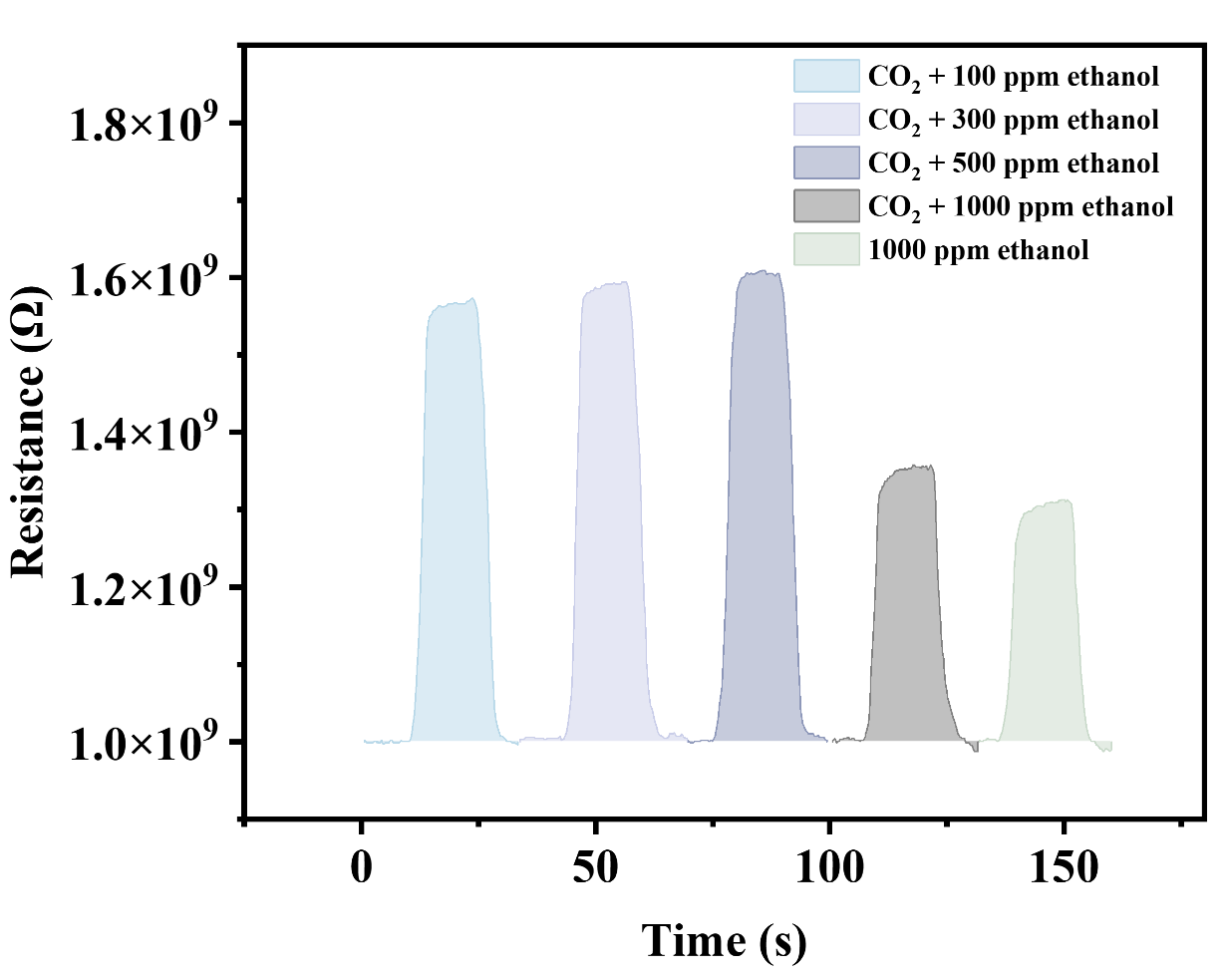


Figure S9. Response curves of FeNC/MoS_2_-1:2 at 100 ppm CO_2_ with different concentrations of ethanol.


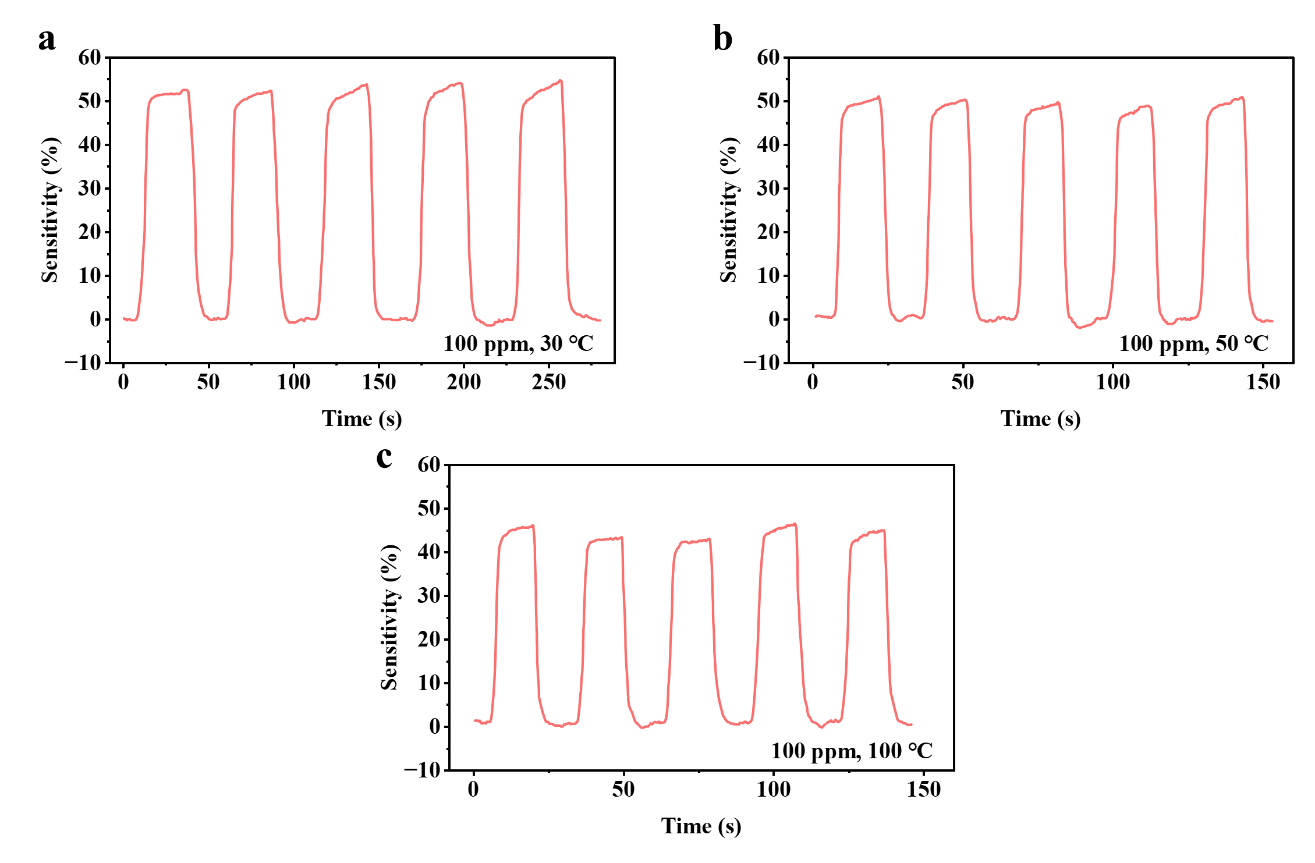
Figure S10. Response curves to 100 ppm CO_2_ of repeatability tests at (a) 30 ℃, (b) 50 ℃, and (c) 100 ℃.


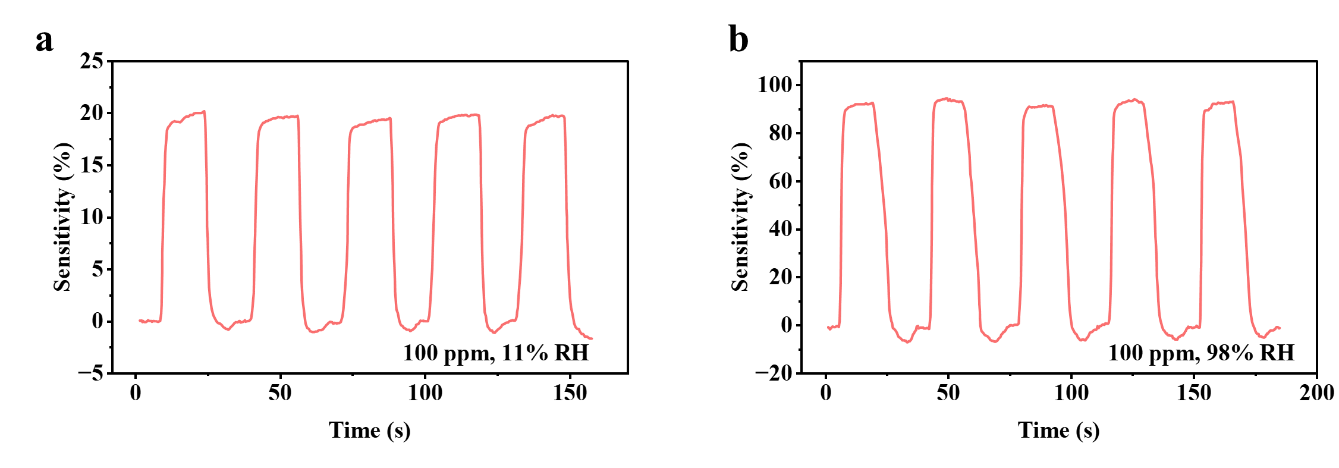


Figure S11. Response curves to 100 ppm CO_2_ of repeatability tests at (a) 11% RH and (b) 98% RH.


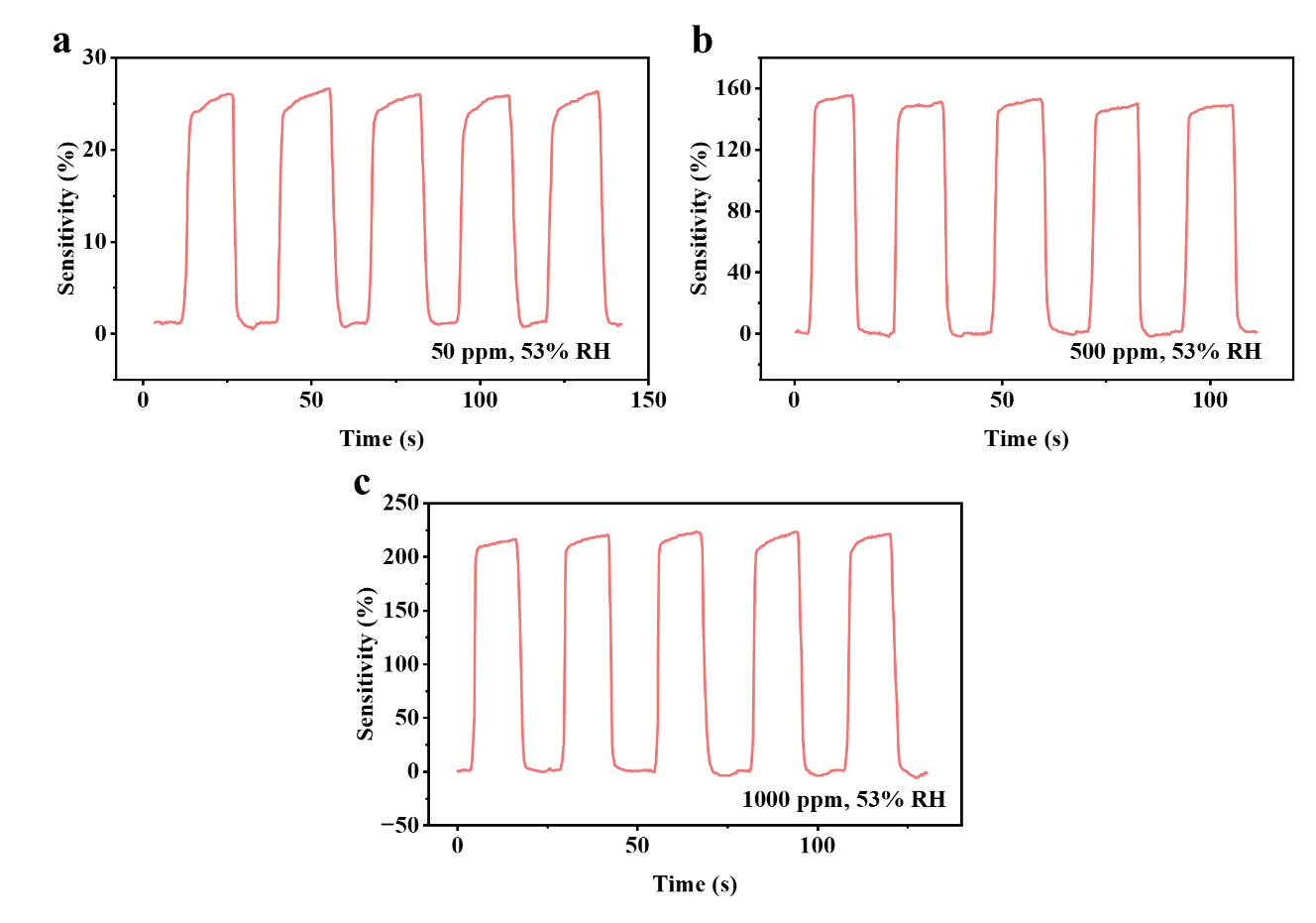
Figure S12. Response curves of repeatability tests for CO_2_ at (a) 50 ppm, (b) 500 ppm, and (c) 1000 ppm.


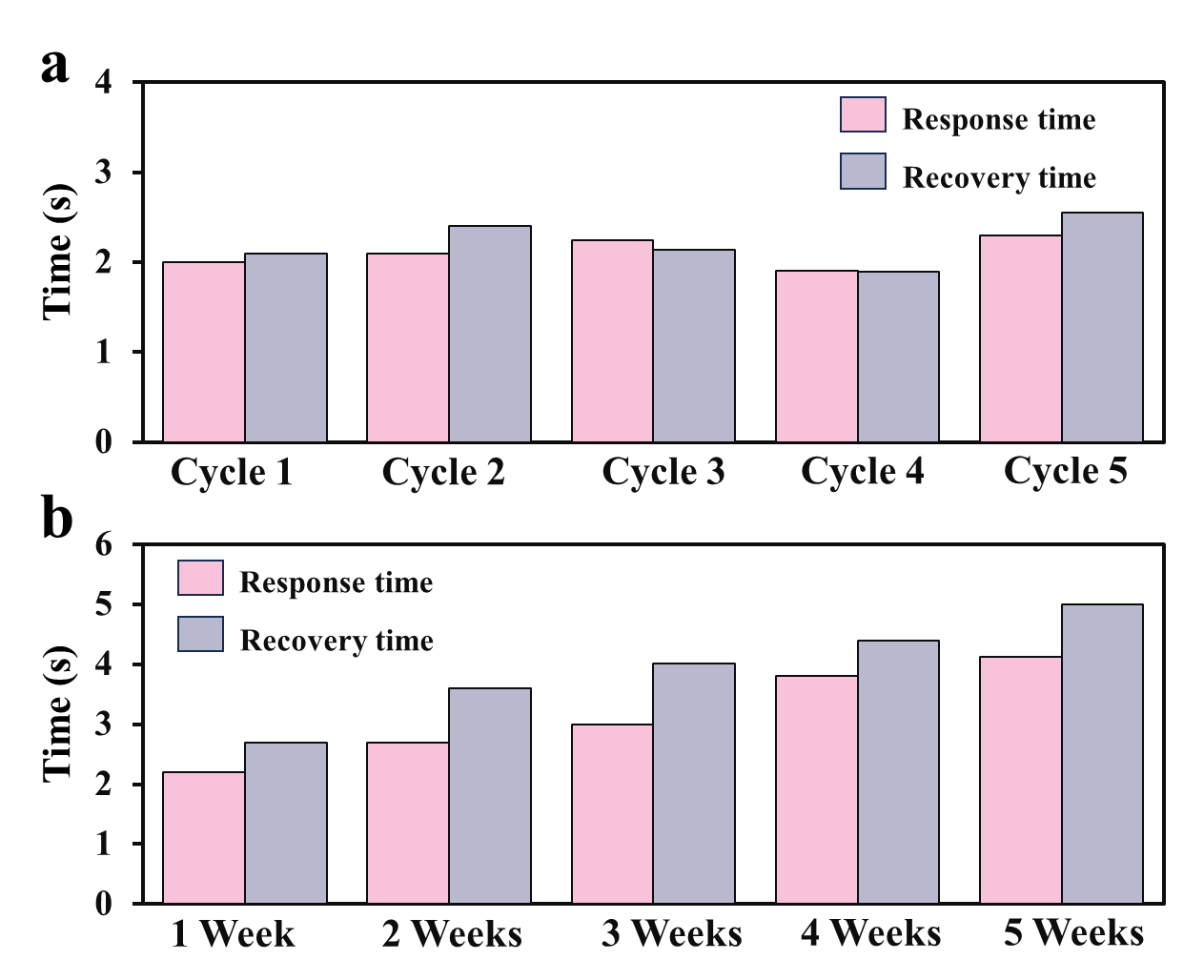


Figure S13. Response and recovery times of the sensor shown in Figures 3i and 3j.


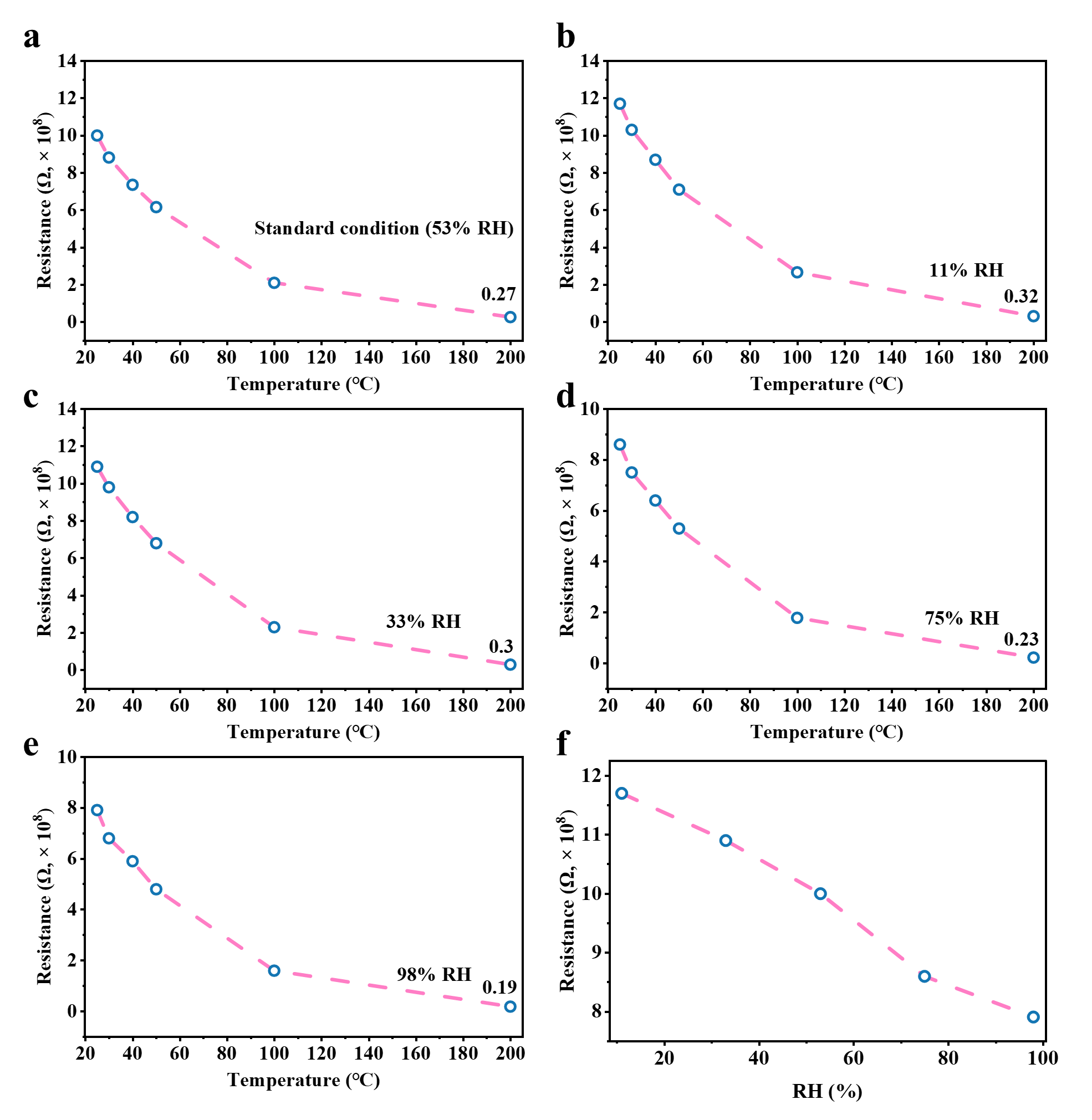


Figure S14. Resistance changes with temperature under different RHs. (a)53%, (b)11%, (c) 33%, (d) 75%, and (e) 98%. (f) Resistance changes with RH under atmospheric conditions.


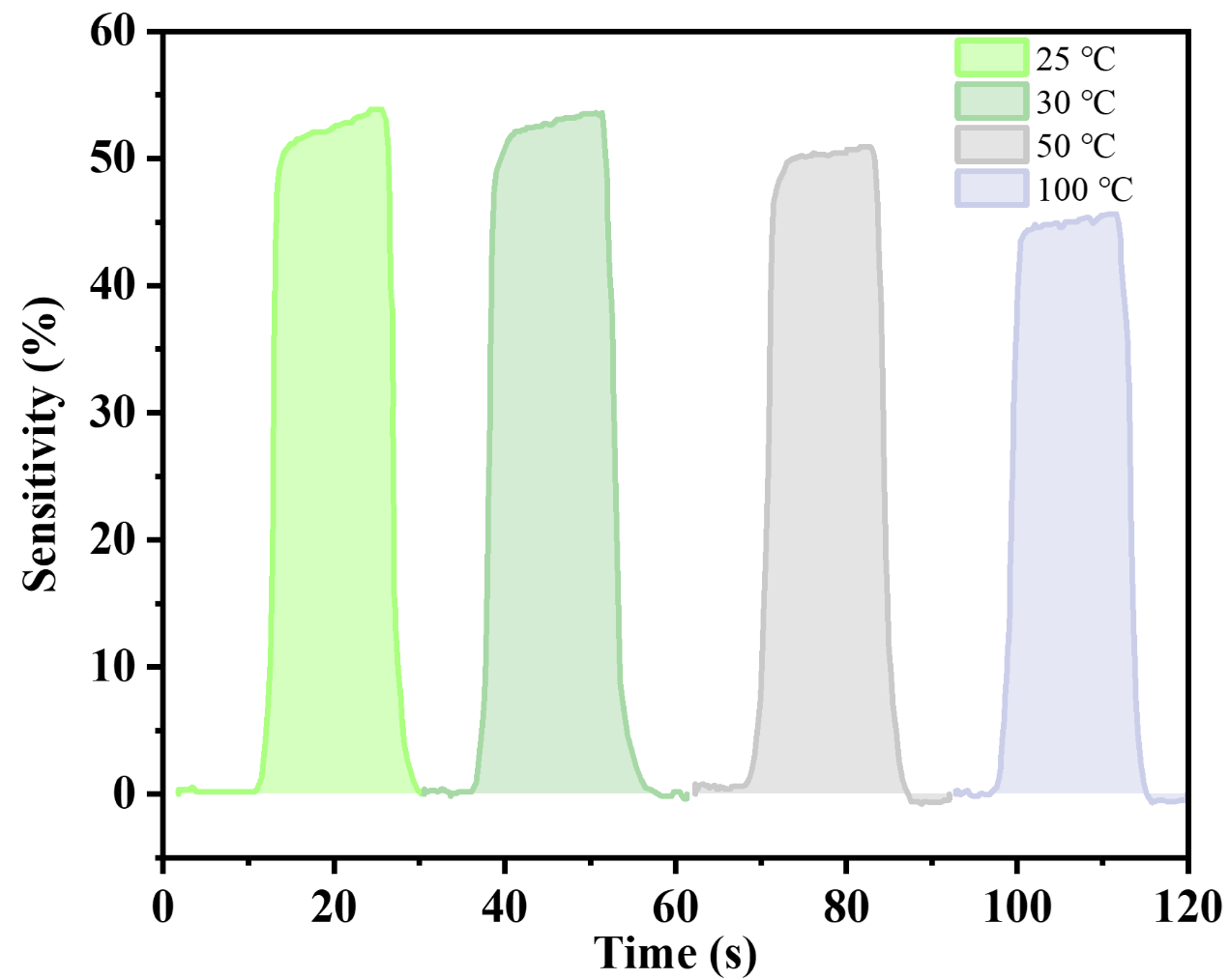


Figure S15. Response curves of FeNC/MoS_2_-1:2 at 100 ppm CO_2_ under different temperatures.


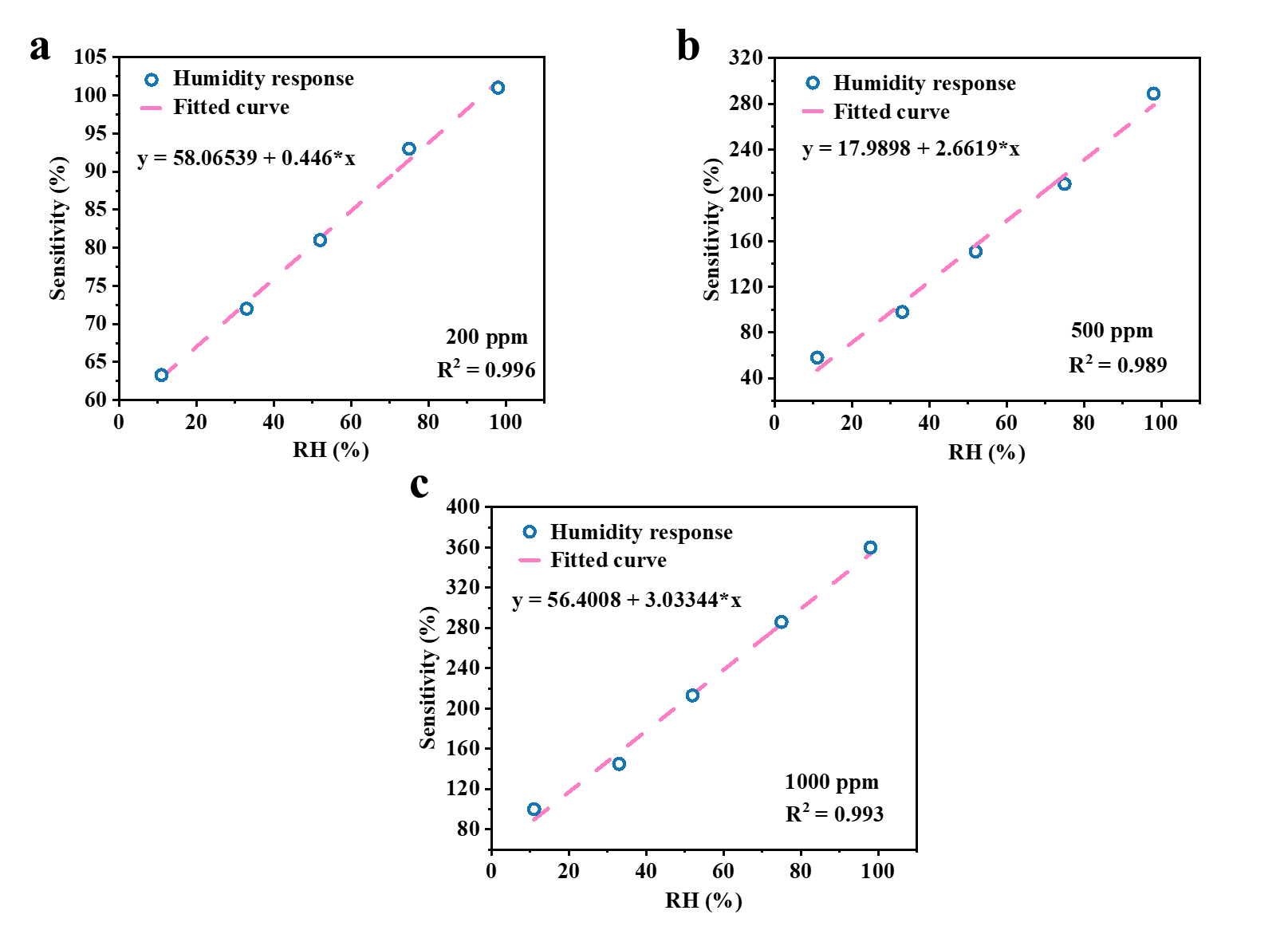


Figure S16. Response curves of FeNC/MoS_2_-2:1 at different CO_2_ concentrations in different RH (a) 200 ppm, (b) 500 ppm, and (c) 1000 ppm.

Figure S17. Pure humidity sensitivity in the absence of CO_2_  under standard conditions.


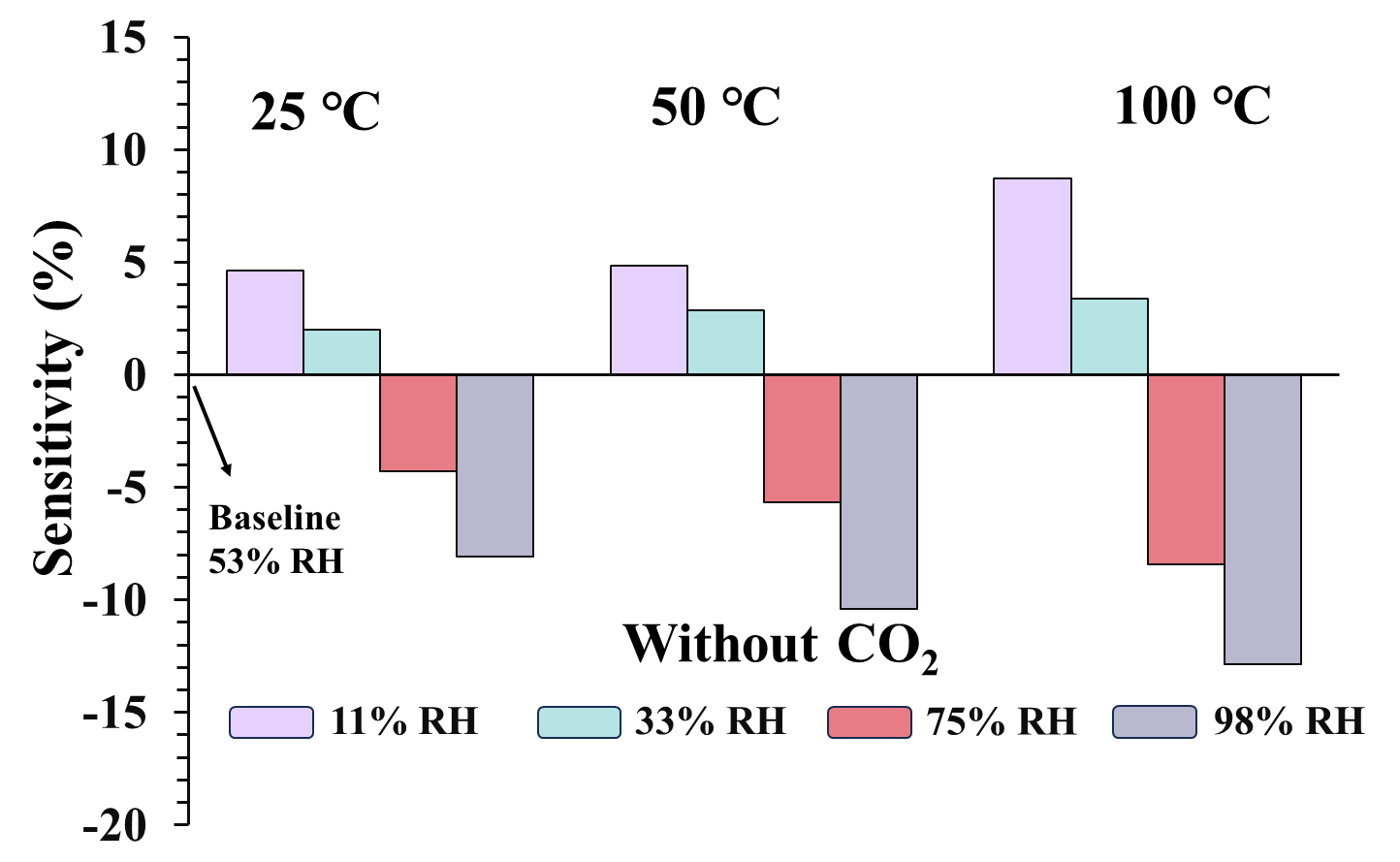


Figure S18. Sensitivity of humidity at different temperatures under synthetic air (53% RH).


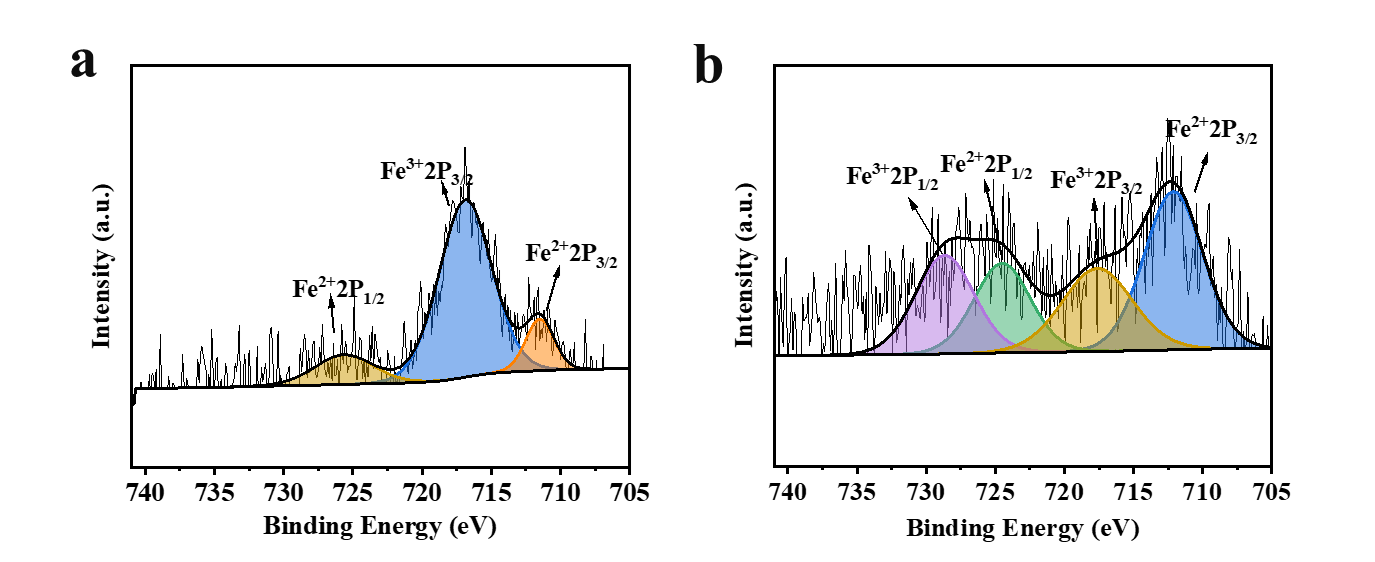


Figure S19. XPS spectra of (a) FeNC and (b) FeNC/MoS₂-1:2.


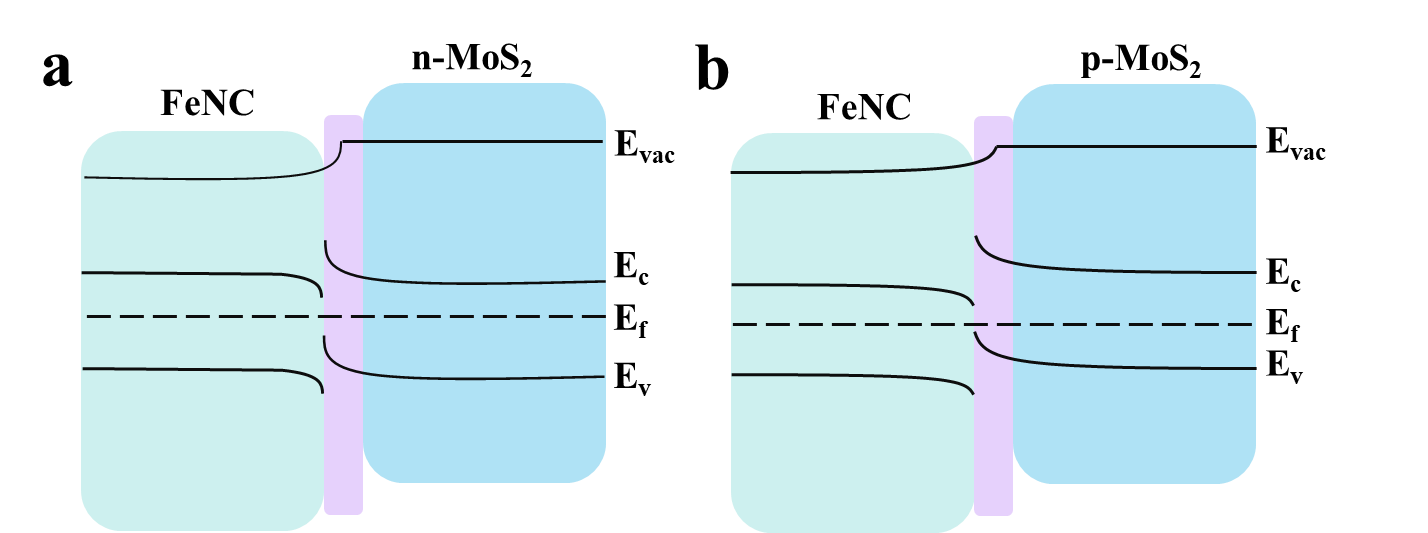


Figure S20. Diagram of energy band (a) n-type FeNC/MoS_2_ and (b) p-type FeNC/MoS_2_.


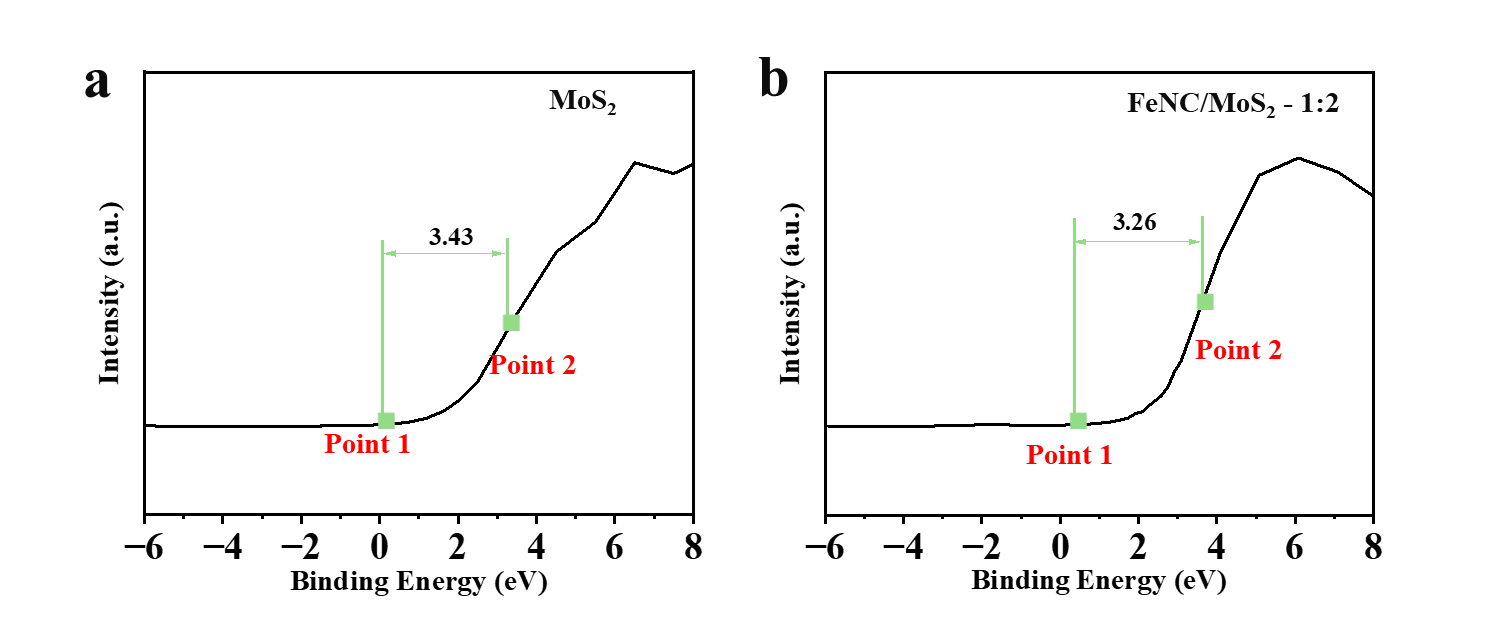


Figure S21. Work function of (a) MoS₂ and (b) FeNC/MoS₂-1:2.


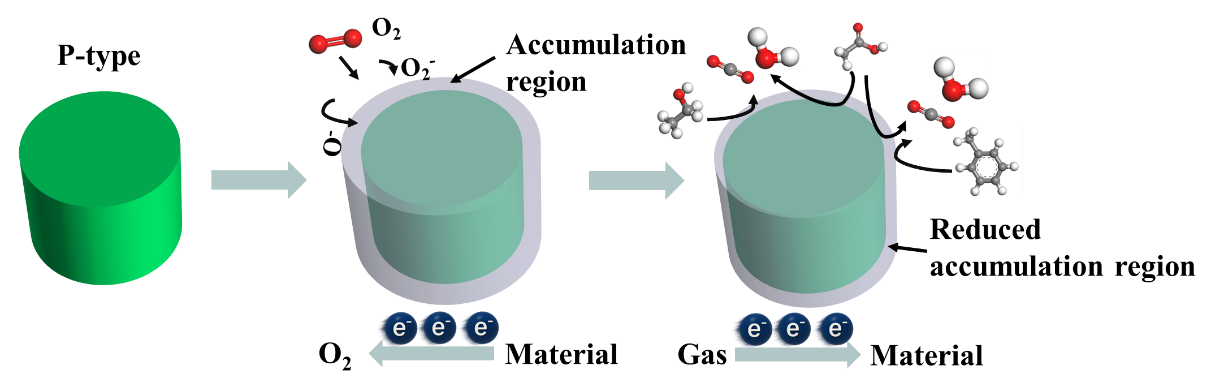


Figure S22. Sensing mechanism of p-type FeNC/MoS_2_ in the interfering gases..


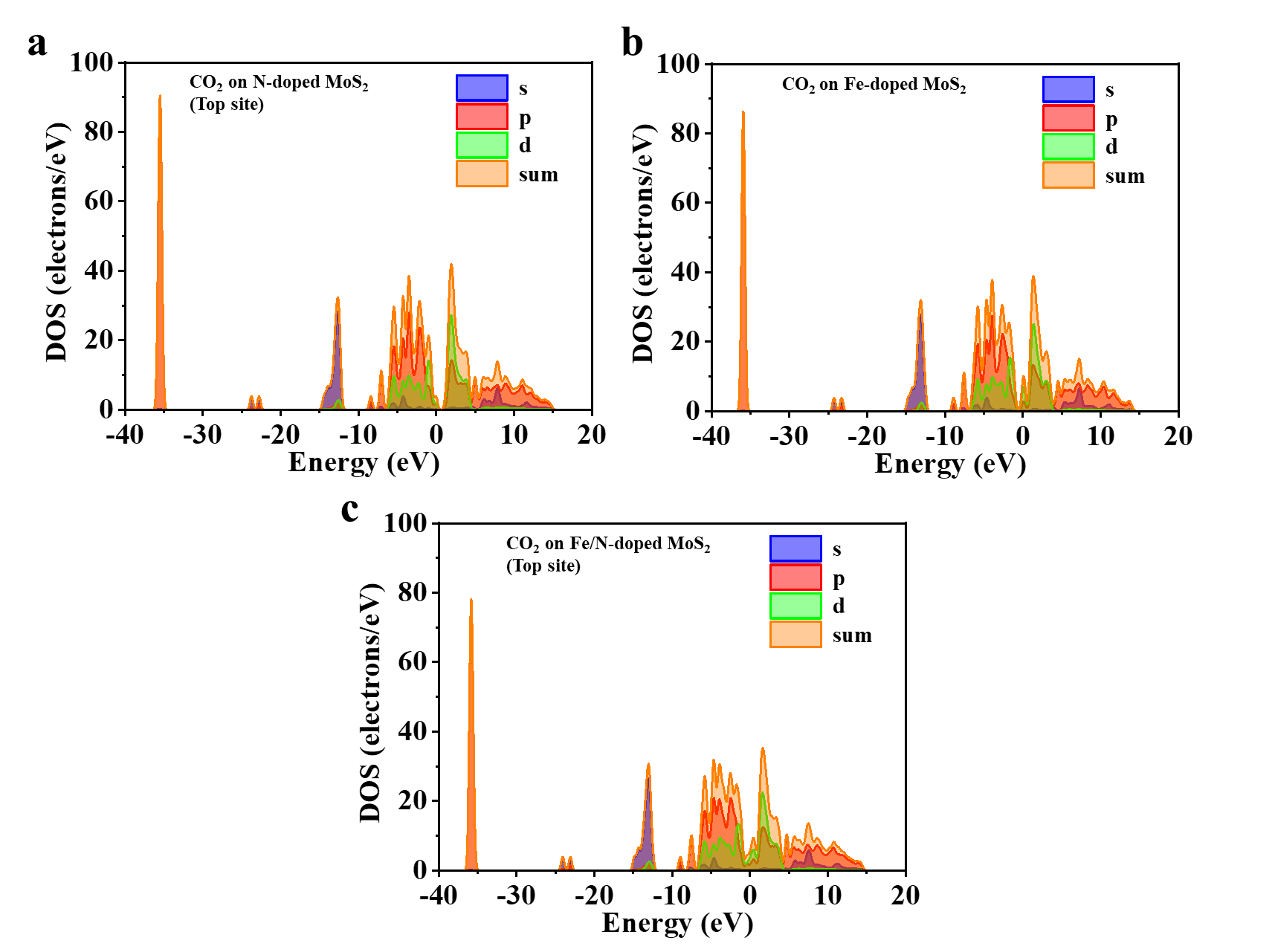


Figure S23. DOS and PDOS of CO_2_ with (a) N-doped MoS_2_, (b) Fe-doped MoS_2_, and (c) Fe/N-doped MoS_2_.


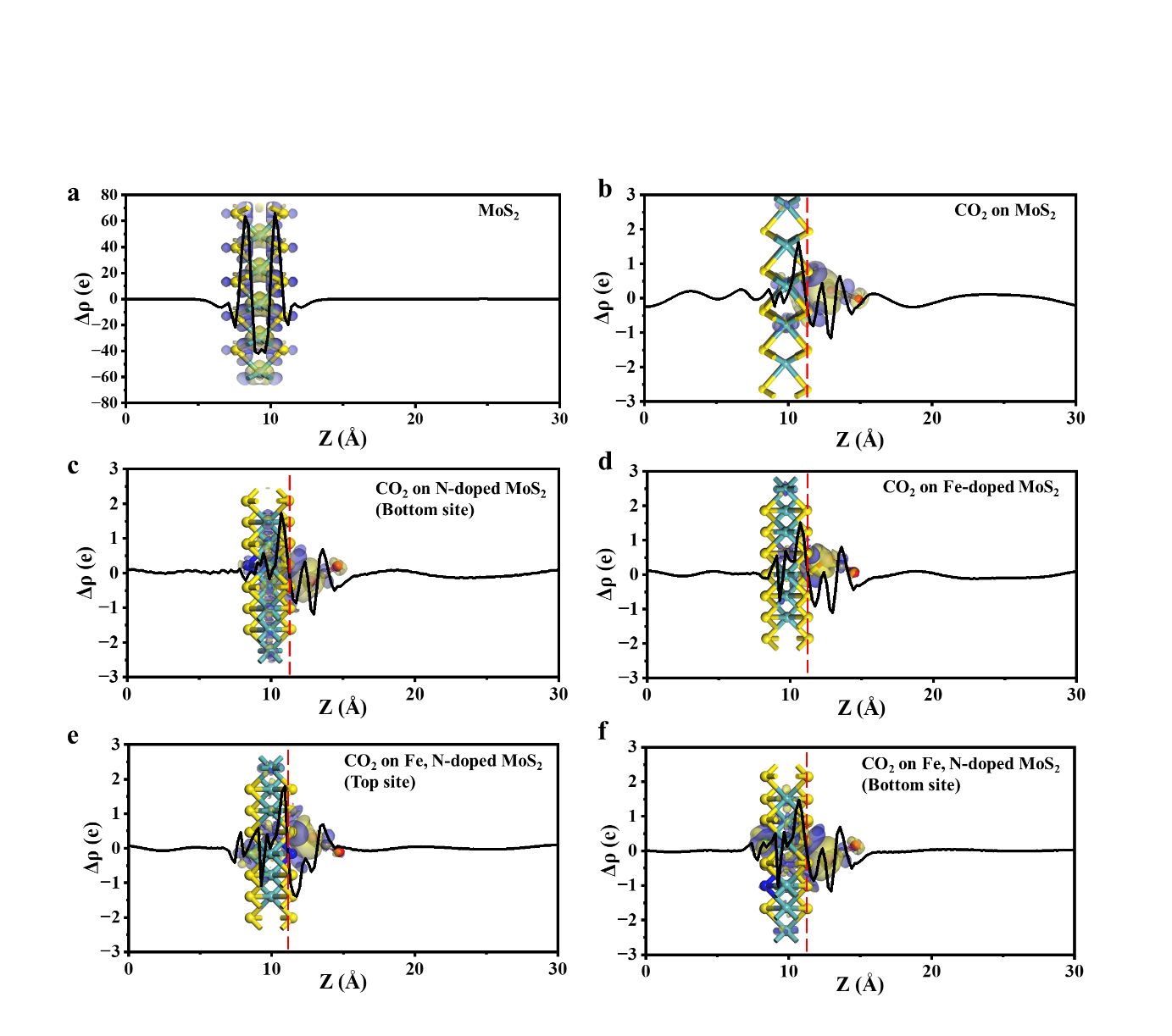


Figure S24. Statistics of differential charge along the Z-axis of (a) MoS_2_, (b) CO_2_ on MoS_2_, (c) CO_2_ on N-doped MoS_2_, (d) CO_2_ on Fe-doped MoS_2_, (e-f) CO_2_ on Fe, N-doped MoS_2_.


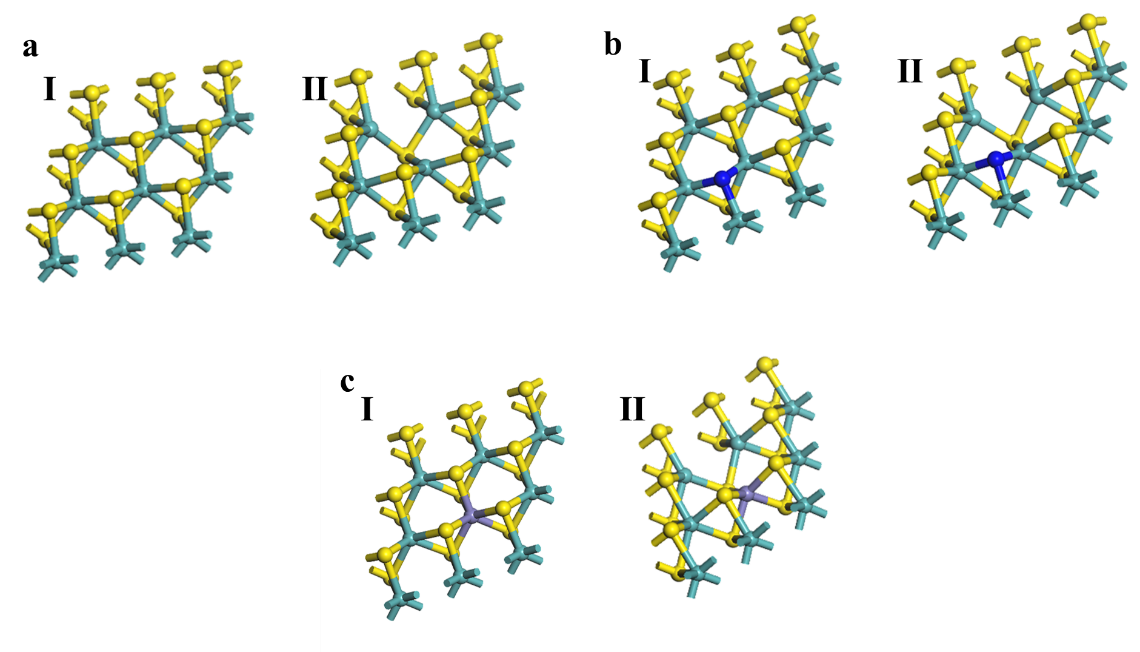


Figure S25. Visualized models for DFT calculations of vacancy formation energies of (a) S, (b) N, and (c) Fe.


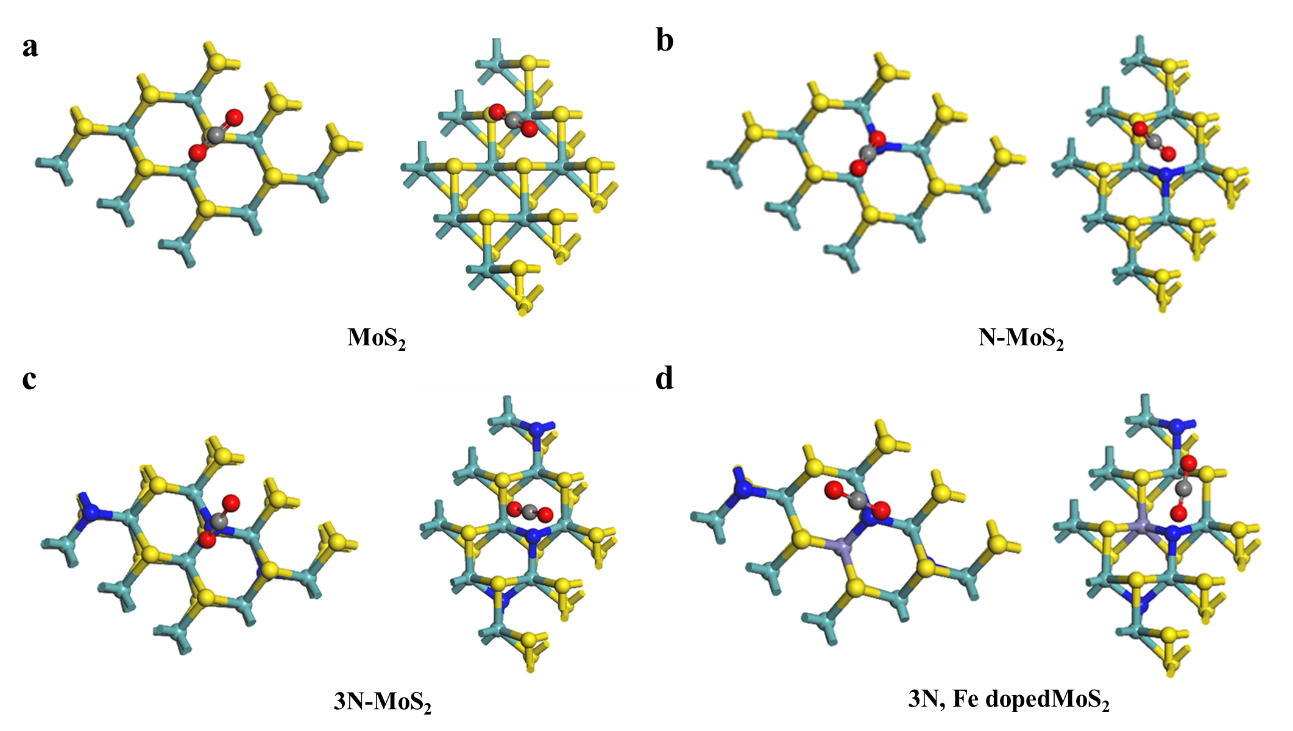


Figure S26. (a-d) Visualized models for DFT calculations of CO_2_ on different materials.


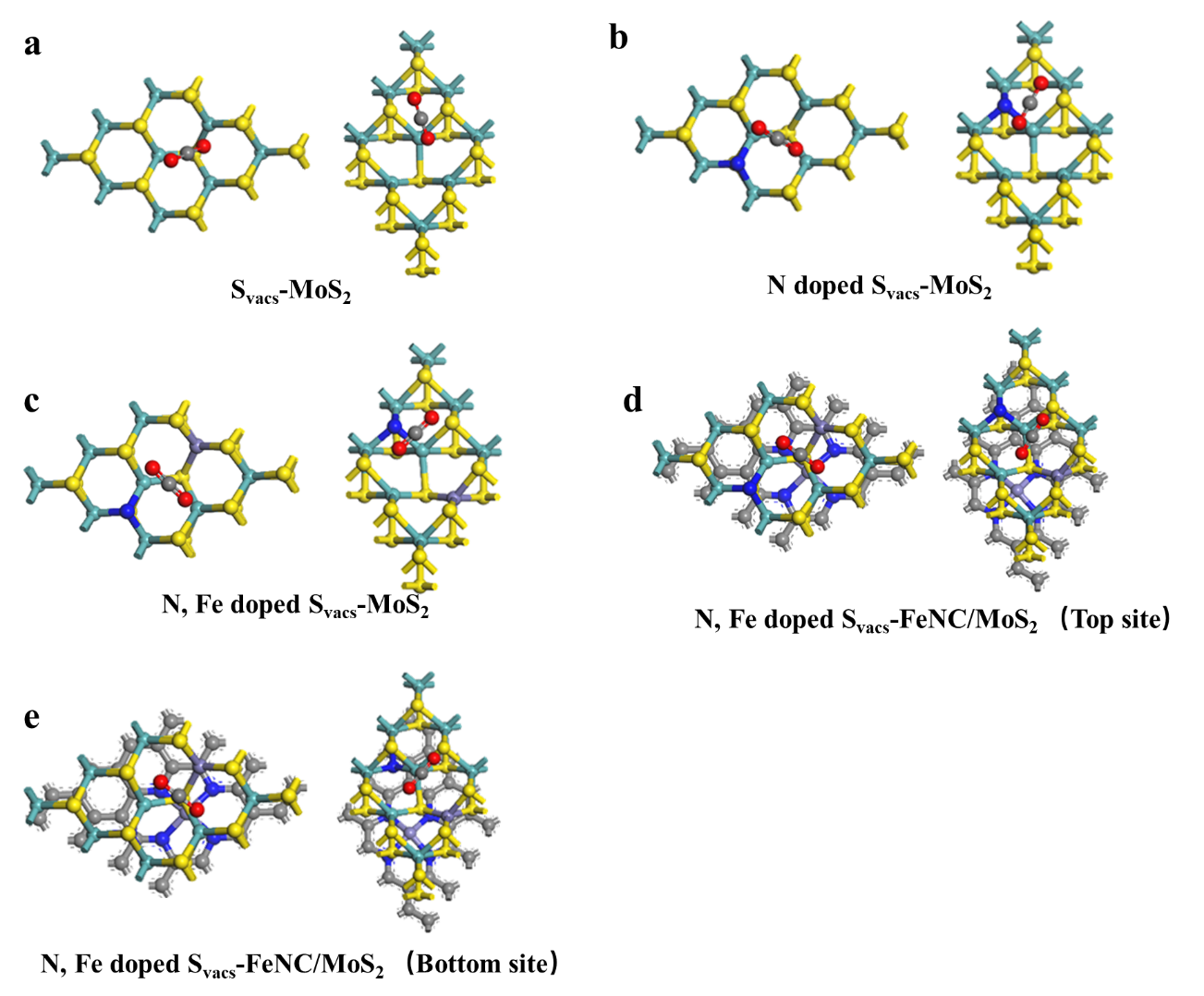


Figure S27. (a-e) Visualized models for DFT calculations of CO_2_ on different materials.


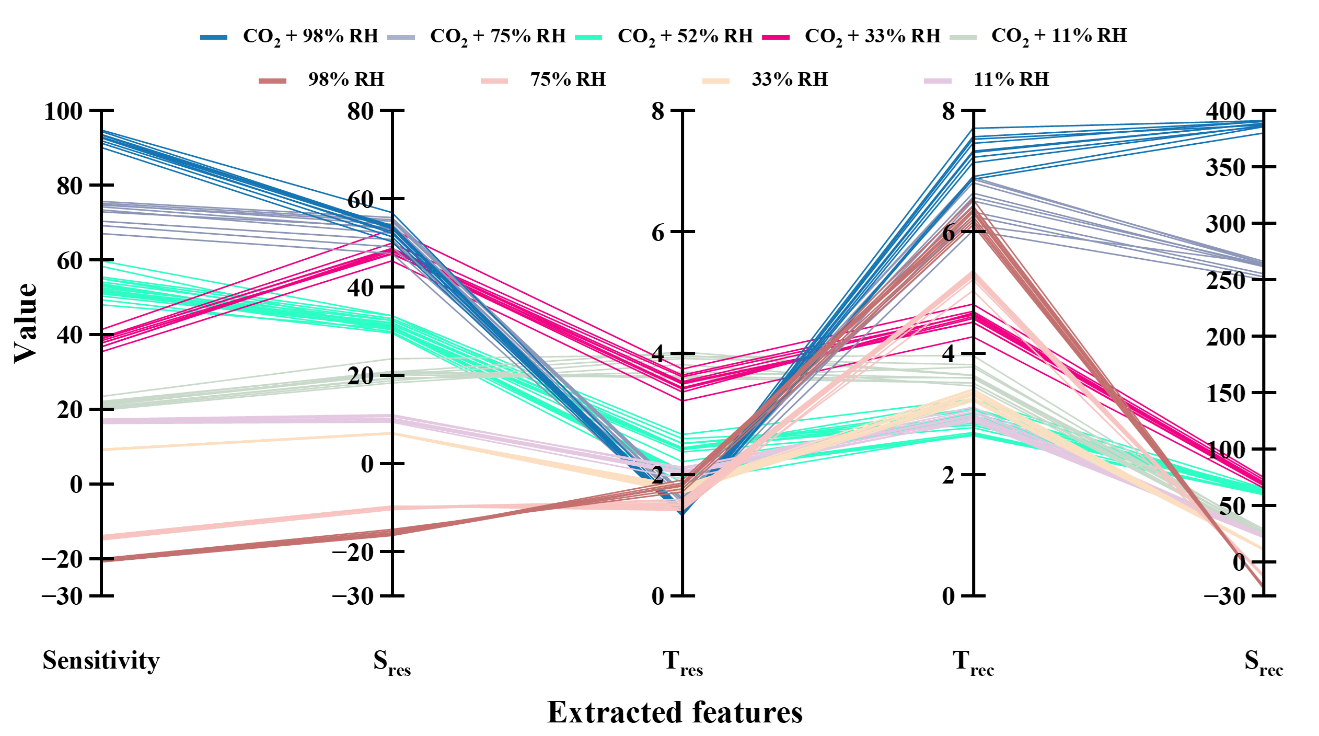


Figure S28. Parallel coordinate plot of all extracted features.


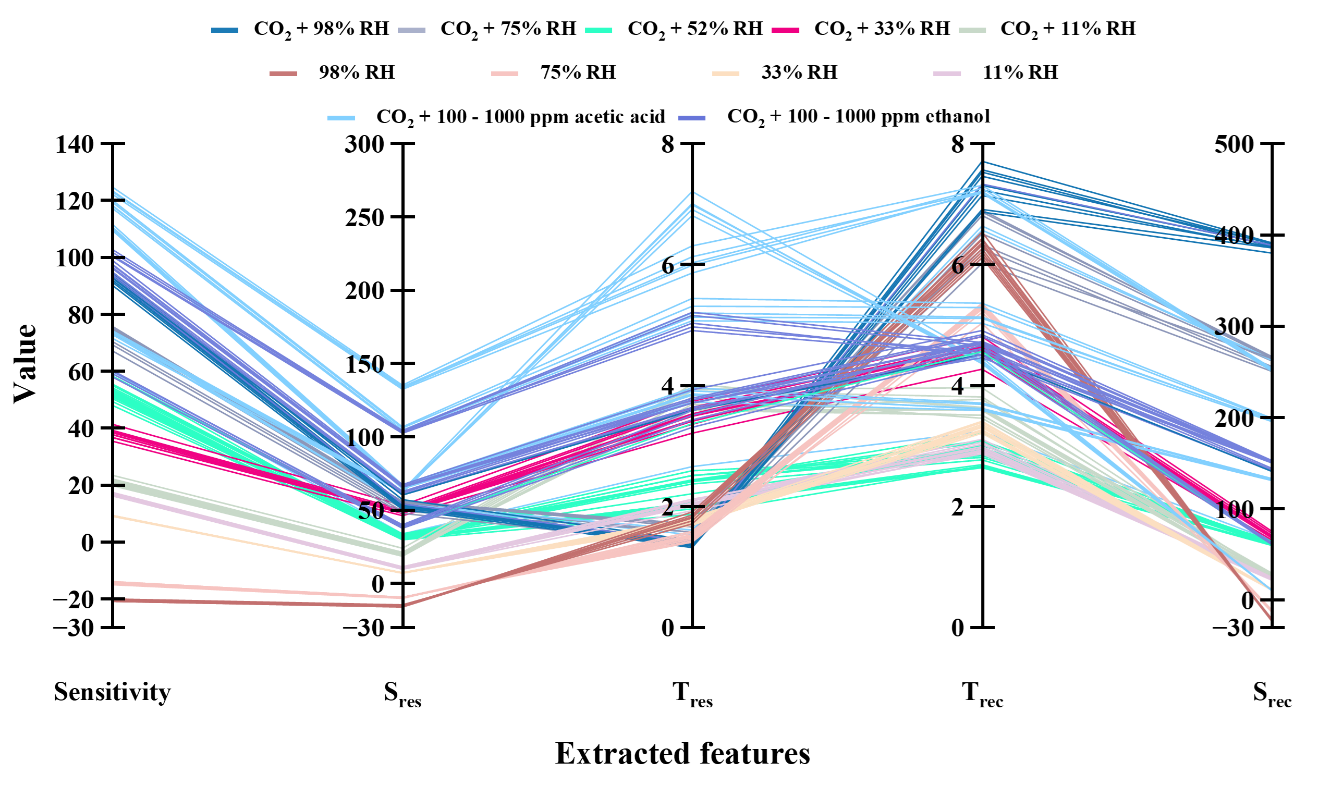


Figure S29. Parallel coordinate plot of all extracted features.


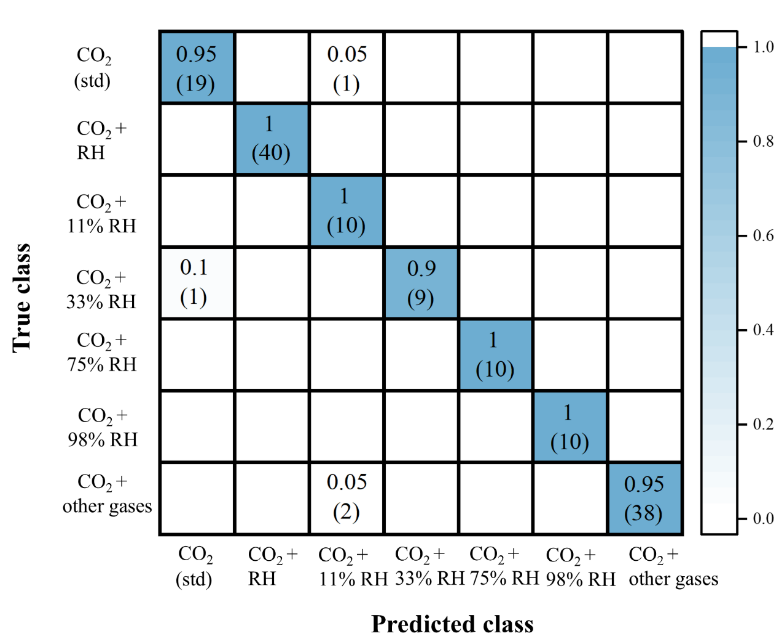


Figure S30. Confusion matrix for seven different groups. The standard condition (std): 100 ppm CO_2_, 53% RH and 25℃.


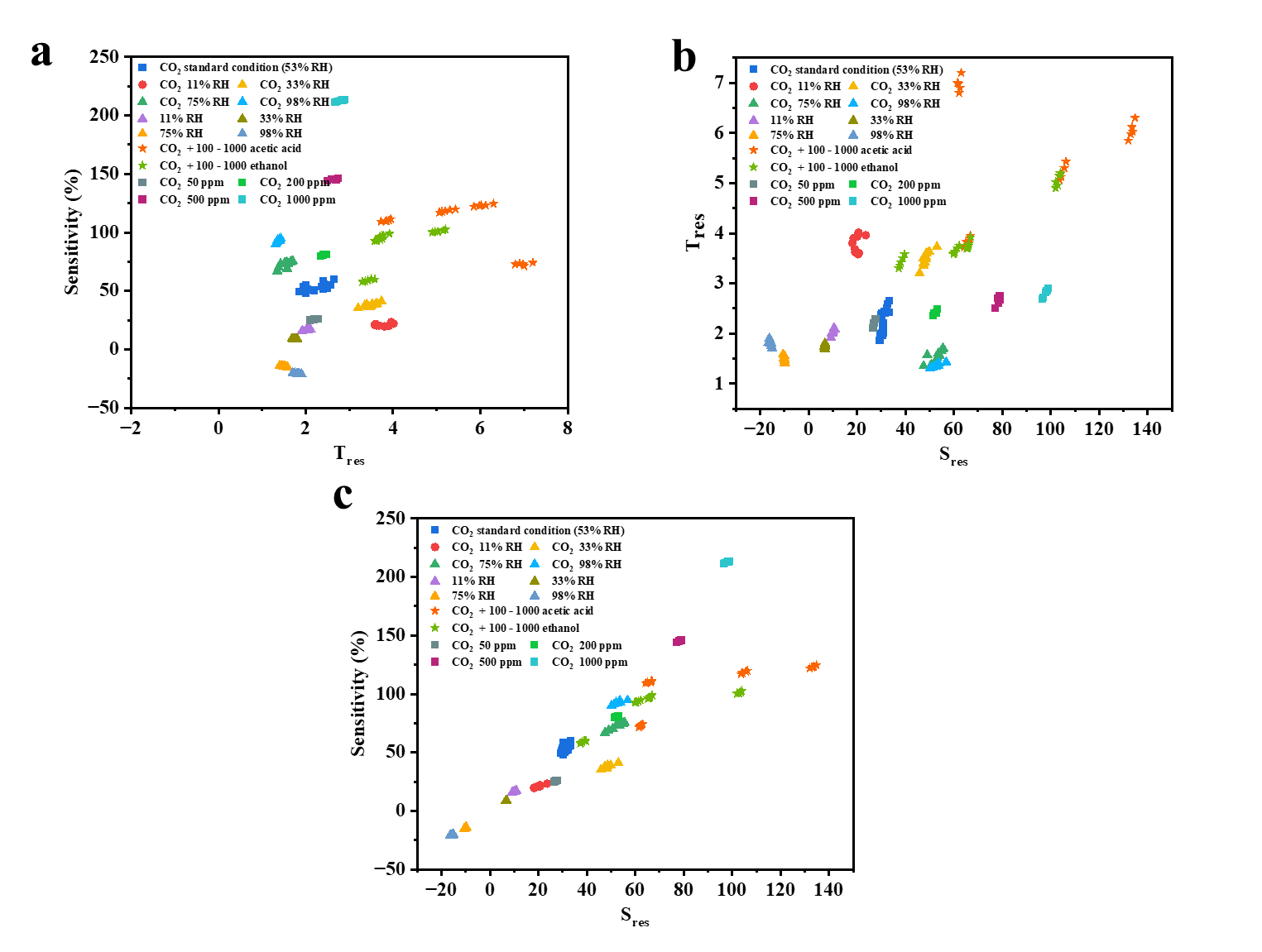


Figure S31. Two-dimensional projection plots (a) T_res_ and sensitivity, (b) S_res_ and T_res_, and (c) S_res_ and sensitivity.


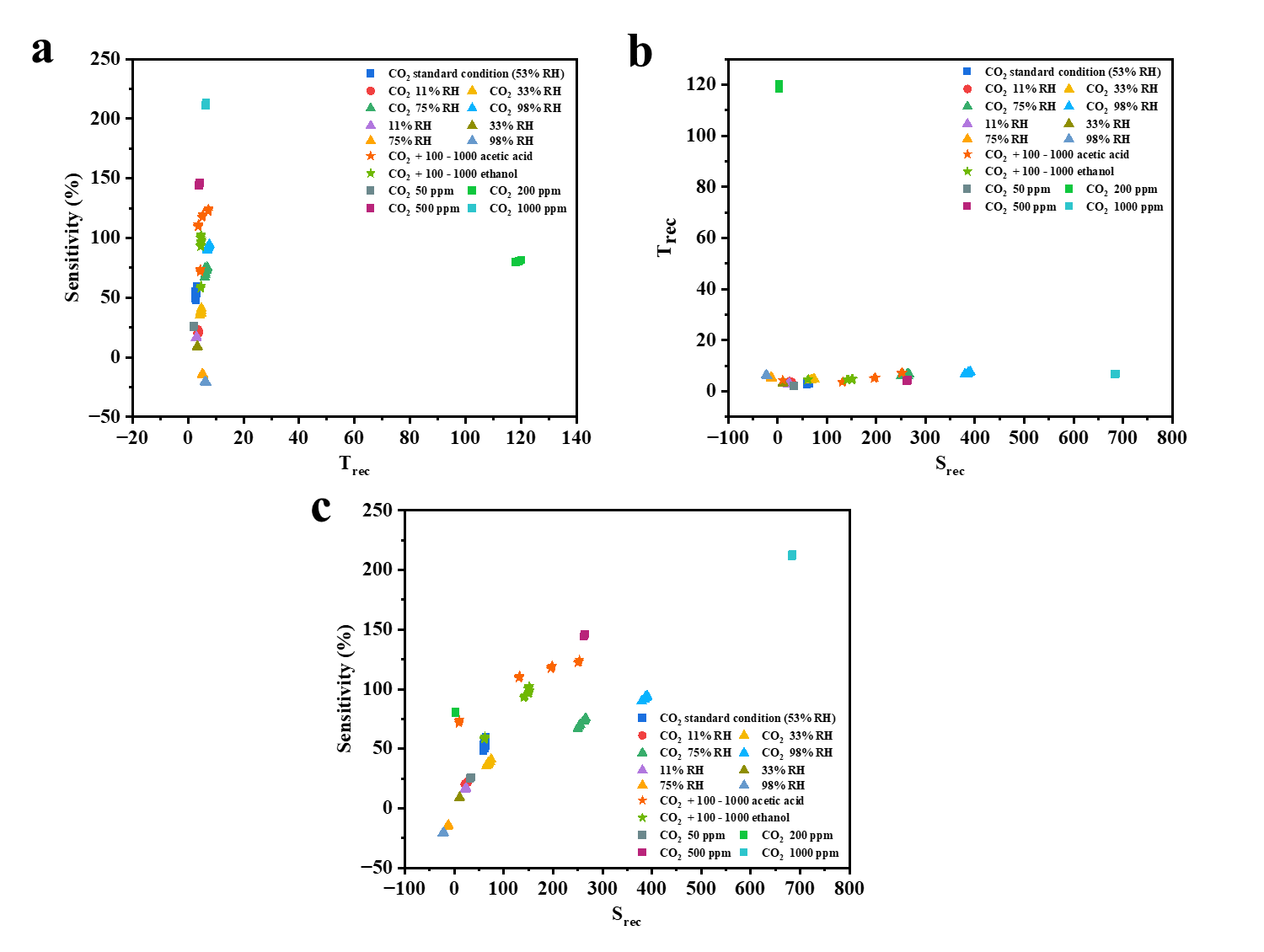


Figure S32. Two-dimensional projection plots (a) T_rec_ and sensitivity, (b) S_rec_ and T_rec_, and (c) S_rec_ and sensitivity.

Table S1 Comparison of adsorption energies for N- and Fe-doped S_vacs_-FeNC/MoS₂, and the sensitivity of FeNC/MoS₂-1:2.

| Gas | E_ads_ (eV) | (E_ads,_ co_2_ - E_ads, gas_)/E_ads,_ co_2_ × 100% | Sensitivity (%, 200 ppm) | (Sco_2_ - S_gas_)/ Sco_2_ × 100% |
| --- | --- | --- | --- | --- |
| carbon dioxide | -2.27 |  | 79.0% |  |
| acetic acid | -0.79 | 65.2% | 20.2% | 74.4% |
| ethanol | -0.68 | 70.0% | 12.6% | 84.1% |
| ammonia | -0.52 | 77.1% | 20.6% | 73.9% |
| acetone | -0.38 | 83.3% | 12.8% | 83.8% |
| formaldehyde | -0.3 | 86.8% | 10.7% | 86.5% |
| toluene | -0.26 | 88.5% | 7.6% | 90.4% |

Table S2. Calculation results of adsorption energy.

| E_ads_ (eV)  Models | CO_2_ on the top site | CO_2_ on the bridge site |
| --- | --- | --- |
| N, Fe doped S_vacs_-FeNC/MoS_2_ | N:-1.8 | Fe-S:-1.4 |
|  | Fe: -1.3 | Mo-S:-1.1 |
|  | S_vacs_: -2.63 | Mo-N:-1.9 |
|  | Mo:-1.0 |  |
|  | S: -0.4 |  |
| N, Fe doped S_vacs_-MoS_2_ | N:-1.6 | Fe-S:-1.3 |
|  | Fe:-1.5 | Mo-S:-1.0 |
|  | S_vacs_: -2.27 | Mo-N:-1.7 |
|  | Mo:-0.9 |  |
|  | S:-0.3 |  |

We summarized the DFT results alongside the experimental values in Table R2. The calculated adsorption energy decreased from a maximum of –2.27 eV for CO₂ to –0.26 eV for toluene, a change of 88.5%. Experimentally, the sensitivity dropped from 79% for CO₂ to 7.6% for toluene, a change of 90.4%. Overall, the computational results were in good agreement with the experimental findings.

**Discussion of TEM results**

We observed that the deposited MoS₂ phase on FeNC nanosheets primarily formed up to two layers, with an average interlayer spacing (d) of 0.59 ± 0.02 nm (Figure S6). At a precursor mass ratio of 1:2, three to four layers were formed (d ≈ 0.63 ± 0.02 nm) (Figures 2d-2h), and more than ten layers were observed at the ratio of 1:6 (d ≈ 0.65 ± 0.02 nm). As the MoS₂ content increased, the interlayer spacing gradually expanded. According to previous reports, the interlayer spacing of 2H-MoS₂ was approximately 0.65 nm [1,2]. When the composite exhibited p-type behavior, N substituted for S atoms. Due to the atomic size difference between N and S, lattice contraction occurred, resulting in a reduced interlayer spacing. However, as the MoS₂ content increased, the limited amount of dopant could no longer convert the newly formed MoS₂ into the p-type phase, leading to a gradual increase in interlayer spacing, approaching that of pristine 2H-MoS₂.

**Distinct response behaviors of FeNC and n-MoS₂**

Due to the electron-rich characteristics of both FeNC and n-MoS₂, their interaction with CO₂ was primarily dominated by chemisorption. Generally, CO₂ reacted with ionized oxygen on the material surface to form $\mathrm{CO}_{3}^{2-}$ species. These $\mathrm{CO}_{3}^{2-}$ were highly unstable and decomposed rapidly, releasing electrons back into the conduction band, thereby increasing the concentration of free charge carriers and resulting in a decrease in resistance. On the other hand, CO₂ could also undergo a reduction reaction by capturing electrons and producing CO molecules. This process reduced the concentration of free charge carriers, leading to an increase in resistance. These two competing reaction pathways tended to offset each other, resulting in a relatively small overall change in resistance. Notably, FeNC significantly lowered the energy barrier for the reduction of CO₂ to CO, making the CO₂ reduction reaction the dominant pathway in the FeNC system, which ultimately manifested as an increase in resistance. In contrast, in the case of n-MoS₂, the reaction of ionized oxygen with CO₂ to form $\mathrm{CO}_{3}^{2-}$ was more favorable, leading to a dominant response of resistance decrease.

**Resistance baseline drift**

For Pristine FeNC and MoS₂: At this stage, CO₂ molecules primarily interact with O²⁻ ions on the material surface, forming a charge-trapping effect. This interaction is relatively strong, and part of the electron transfer cannot be fully reversed in a short time, resulting in the resistance after the gas response not fully returning to its initial state.

For n-ytpe FeNC/MoS₂ (1:8): It exhibits typical n-type behavior in gas sensitivity, with a mechanism similar to that of pristine FeNC and MoS₂, and therefore also cannot achieve complete recovery.

For p-type FeNC/MoS₂ (1:0.5 and 1:2): CO₂ molecules are primarily adsorbed and desorbed through interactions with defect sites (vacancies). Because this mechanism is relatively weak and reversible, complete resistance recovery was achieved at a 1:2 component ratio. However, at a 1:0.5 ratio, the thinner MoS₂ layers may not fully cover the FeNC, allowing part of the FeNC to participate in the reaction, resulting in incomplete resistance recovery. It is noteworthy that when the recovery time is sufficiently long, nearly all samples can return to their initial resistance.

**Repeatability and long-term stability**

As shown in Figure S13, the response and recovery times fluctuated only slightly during the cycling tests. However, in the long-term repeatability tests, both response and recovery times increased with prolonged exposure. To simulate material stability under real-use conditions, the device was placed in an open environment without any protective measures, with temperature and humidity matching the ambient conditions and exposed to light. As a result, the material surface may have been contaminated or oxidized, leading to longer response and recovery times. Moreover, the increase in recovery time was greater than that of the response time, likely due to strong interactions between contaminants and CO₂, which slowed desorption. Therefore, device encapsulation is necessary to protect the sensitive material.

**Conductivity**

For n-type MoS₂/FeNC, due to the work function difference, electrons transfer from MoS₂ to FeNC, which is consistent with theoretical calculations. As electrons flow out, an electron-depletion region forms on the n-type MoS₂ surface, causing both the valence band (E_v_) and conduction band (E_c_) to bend upward relative to the Fermi level (E_f_). Meanwhile, FeNC, acting as an electron acceptor, exhibits a slight downward band bending. Eventually, the E_f_ of both materials align, and the vacuum level (E_vac_) bends correspondingly to maintain the relative energy relationship between the bands (Figure S20a).

For p-type MoS₂/FeNC, although the work function of p-type MoS₂ is higher than that of n-type MoS₂, it is still lower than that of FeNC. Therefore, electrons transfer from p-MoS₂ to FeNC, increasing the hole concentration on the p-MoS₂ surface and forming a hole-rich layer. The E_v_ and E_c_ of p-MoS₂ bend upward relative to the E_f_, while FeNC, as an electron acceptor, still exhibits downward band bending. The E_f_ of both sides eventually align, and the E_vac_ bends accordingly to preserve the relative energy relationships between bands. Difference between n-type and p-type MoS₂/FeNC: Because the work function of p-type MoS₂ is higher than that of n-type MoS₂, its E_c_ is closer to the vacuum level. The reduced work function difference with FeNC results in fewer electrons transferring from p-type MoS₂ to FeNC compared to n-type MoS₂. Consequently, the band bending in p-type MoS₂ within the composite is smaller than that of n-type MoS₂ (Figure S20b).

Electron transfer in sensing behavior: For FeNC, pristine MoS₂, and n-type MoS₂/FeNC, the gas sensing mechanism is the same. In air, O₂ molecules are adsorbed onto the sensing layer surface and capture electrons from the conduction band to form surface oxygen anions (O⁻, O₂⁻, and O²⁻). This process induces a depletion layer on the n-type semiconductor surface, increasing the resistance. Upon exposure to the target gas, the adsorbed oxygen anions react with gas molecules, releasing electrons back to the E_c_, thereby reducing the depletion layer thickness and restoring the resistance (main text, Figure 4j).

For p-type MoS₂/FeNC, the O₂ adsorption and ionization process similarly consumes electrons, decreasing the surface electron density and increasing hole concentration, forming a hole-rich layer and lowering the resistance. When exposed to the target gas, the adsorbed oxygen anions react with gas molecules and release electrons, weakening the hole-rich effect, reducing the thickness of the enriched layer, and restoring the resistance (Figure S22).

**Humidity influence**

According to Figures S17 and S18, water molecules contribute to the sensor signal not only independently but also synergistically with the CO₂ response, which apparently enhances the sensitivity in sensing CO₂. Specifically, at low to moderate humidity levels, water molecules compete with CO₂ for adsorption. The pre-adsorbed water modulates the local charge distribution, which may further convert CO₂ into bicarbonate/carbonate species, amplifying the CO₂ response. As humidity increases (from 53% to 75% RH), physically adsorbed water can form a hydrogen-bond network, and the proton conduction effect gradually dominates [3, 4]. At this stage, the interaction between water and CO₂ is further enhanced, leading to additional amplification of the response. When humidity rises further (from 75% to 98% RH), the adsorption of water and its interaction with CO₂ reach saturation, and the amplification of the response slows down. Moreover, in the absence of CO₂, the effect of humidity on sensitivity is significantly lower than in the presence of CO₂, further confirming the interaction between water and CO₂. Overall, part of the measured CO₂ sensitivity arises from water-induced competing adsorption and proton conduction effects, rather than solely from the intrinsic interaction between CO₂ and the sensor.

In Figure S18, it can be seen that in the absence of CO₂, the contribution of humidity to sensitivity increased with temperature in the 25-100 °C range. Moreover, when the temperature rose from 50 °C to 100 °C, the change in sensitivity was larger than that from 25 °C to 50 °C, which may be attributed to more pronounced resistance changes caused by water molecule dissociation or the increased likelihood of ion channel formation.

**Doping and CO_2_ adsorption**

In MoS₂ systems without vacancies, N and Fe doping also effectively enhanced the adsorption capacity for CO₂. Because electrons from the MoS₂ matrix tended to migrate toward the N and Fe dopant sites, making these sites more negatively charged. When a CO₂ molecule approached, its oxygen atoms could partially transfer electrons to the N/Fe-doped sites, resulting in a stronger interaction and more stable binding. Additionally, the spatial positional differences between the N and Fe dopants also affected the adsorption performance. Typically, N atoms that located closer to the MoS₂ surface can induce a stronger local charge accumulation, leading to higher binding energies with CO₂ molecules.

**Surface energy and gas adsorption**

Previous studies have demonstrated that dispersive forces exist universally between all molecules; therefore, an increased number of dispersion component active sites facilitated interactions with gas molecules, especially for non-polar gases [5]. This was because the adsorption of non-polar gases primarily depended on the dispersion forces of the material surface. Conversely, polar forces occurred mainly between polar molecules, with highly polar molecules preferentially adsorbing onto polar sites. Reported polarity orders for various gases are as follows: acetic acid > ammonia > ethanol > acetone > formaldehyde > toluene, which can explain the results shown in Figure 3g. Theoretically, polar gas sensitivity should follow this order, but practical adsorption is also influenced by molecular bond energy, environmental humidity, and other material properties, meaning the sensitivity sequence could not strictly follow this order. Also, when the surface energy of the material exceeded that of the adsorbate molecules, adsorption was facilitated. Overall, an increase in the dispersion component and surface energy was considered favorable for the adsorption of non-polar CO₂.

**Analysis of DOS**

After CO₂ adsorption on N-doped MoS₂, the DOS features appeared near the Fermi level, mainly arising from the hybridization between N 2p and CO₂ O 2p states (Figure S22). This hybridization introduced additional electronic states within the band gap, partially occupying the antibonding orbitals of CO₂ and thereby activating the molecule. Compared with the undoped system, the N-doped sample showed an electronic structure more favorable for CO₂ adsorption and charge transfer. For Fe-doped MoS₂, CO₂ adsorption led to a clear coupling between Fe 3d states and the molecular orbitals of CO₂ (especially O 2p), generating strongly localized d-state peaks at the Fermi level. Although this increased the available electronic states, the relatively localized d states could limit charge-transfer efficiency. Overall, CO₂ could still be adsorbed and exchange electrons, but the activation was less pronounced than in the N-doped or co-doped systems. In the Fe/N co-doped MoS₂, CO₂ adsorption produced an even larger, broader, and more continuous DOS near the Fermi level. Strong hybridization occurred between the p states (from the C and O of CO₂ and the N 2p) and the d states of Fe/Mo, leading to pronounced electronic coupling. As a result, the antibonding orbitals of CO₂ were more readily filled, yielding the strongest charge-transfer and activation effects. Compared with single-doping, the co-doped sample exhibited more stable CO₂ adsorption and enhanced activation capability.

After CO₂ adsorption on N-doped MoS₂, the DOS features appeared near the Fermi level, mainly arising from the hybridization between N 2p and CO₂ of O 2p states. This hybridization introduced additional electronic states in the band gap, partially occupying the antibonding orbitals of CO₂ and thereby activating the molecule. Compared with the undoped system, the N-doped sample showed an electronic structure more favorable for CO₂ adsorption and charge transfer. For Fe-doped MoS₂, CO₂ adsorption led to a clear coupling between Fe 3d states and the molecular orbitals of CO₂ (especially O 2p), generating strongly localized d-state peaks at the Fermi level. Although this increased the available electronic states, the relatively localized d states could limit charge-transfer efficiency. Overall, CO₂ could still be adsorbed and transfer electrons, but the activation was less pronounced than in the N-doped or co-doped systems. In the Fe/N co-doped MoS₂, CO₂ adsorption produced an even larger, broader, and more continuous DOS near the Fermi level. Strong hybridization occurred between the p states (from the C and O of CO₂ and the N 2p) and the d states of Fe/Mo, leading to pronounced electronic coupling. As a result, the antibonding orbitals of CO₂ were more readily filled, yielding the strongest charge-transfer and activation effects. Compared with single doping, the co-doped sample exhibited more stable CO₂ adsorption and enhanced activation capability.

**Collection of exhaled breath**

The exhaled gas samples were collected by the author solely for sensor calibration and performance validation purposes. The study did not involve any medical diagnosis, treatment, or personal data collection; therefore, no ethical approval was required.

Exhaled human breath was collected using a 5 L gas sampling bag, which was evacuated prior to use to remove any residual gases. A gas tube equipped with a valve connected the bag to the mouthpiece. During sampling, the subject placed their mouth tightly against the mouthpiece, opened the valve, and exhaled into the bag five times before closing the valve. For CO₂ concentration testing, a syringe was used to extract a fixed volume of gas from the sampling bag, which was then directly injected into a 20 L rigid gas chamber by piercing a rubber septum with the syringe needle. To prevent gas exchange between the chamber and the environment, the needle was quickly withdrawn, and the puncture site was immediately sealed with adhesive tape. Both the test sensor and a commercial CO₂ sensor were pre-installed inside the chamber. Given that the chamber was made of rigid material and its volume was much larger than that of the syringe, changes in volume and pressure during injection were negligible. The introduced CO₂ concentration (C, %) was calculated using the following equation:

$$C (\%)=\frac{{\Delta C}_{c}\times V_{c}}{10000\times V_{inj}}$$

where ΔC_c_ was the CO₂ concentration change measured in the sampling bag (ppm), V_c_ was the volume of the sampling bag (mL), and V_inj_ was the volume of the gas injected into the chamber (mL).

The CO₂ fluctuation value was obtained using the following equation:

$$Fluctuation \left( \% \right)= \frac{C_{a}}{C_{b}}\times100$$

where C_a_ was the CO_2_ concentration after activities and C_b_ was the CO_2_ concentration before activities.

References

1. Zhang, D. Z.; Jiang, C. X.; Wu, J. F., Sensor Actuat B-Chem 2018, 273, 176-184, 10.1016/j.snb.2018.06.044.

2. Choi, W.; Kim, J.; Lee, H.; Mehta, G.; Prasad, V,. Acs Appl Mater Inter 2021, 13, 11, 13596–13603, 10.1021/acsami.1c00650.

3. Veldhuizen H, Butt S A, Van Leuken A, et al. Competitive and cooperative CO_2_–H_2_O adsorption through humidity control in a polyimide covalent organic framework. ACS Appl. Mater. Interfaces. 2023, 15, 29186-29194, 10.1021/acsami.3c04561.

4. Dhonge B P, Ray S S, Mwakikunga B. Electronic to protonic conduction switching in Cu_2_O nanostructured porous films: The effect of humidity exposure. RSC Adv, 2017, 7, 21703-21712, 10.1039/C7RA00383H.

5. Feinberg, G.; Sucher, J.; Au, C. K., Phys Rep 1989, 180, 83–157, 10.1016/0370-1573(89)90111-7.
